# Synergistic combination of targeted nano-nuclear-reactors and anti-PD-L1 nanobodies evokes persistent T cell immune activation for cancer immunotherapy

**DOI:** 10.1186/s12951-022-01736-8

**Published:** 2022-12-10

**Authors:** Lipeng Zhu, Junnan Li, Ziang Guo, Hang Fai Kwok, Qi Zhao

**Affiliations:** 1 School of Life Sciences, Xiangya School of Medicine, Central South University, Changsha, 510006 China; 2 Cancer Centre, Institute of Translational Medicine, Faculty of Health Sciences, University of Macau, Macau SAR, 999078 China; 3 MoE Frontiers Science Center for Precision Oncology, University of Macau, Macau SAR, China

**Keywords:** Nano-nuclear-reactor, Anti-PD-L1 nanobody, Immunogenic cell death, Hypoxia modulation, T cell immune activation, Immune-cold tumors

## Abstract

**Background:**

Antitumor T cell immunotherapy as a novel cancer therapeutic strategy has shown enormous promise. However, the tumor microenvironment (TME) is characterized by the low immunogenicity, hypoxia, and immunosuppressive condition that dramatically limit effective T cell immunotherapy. Thus, an ideal immunotherapy strategy that is capable of reversing the immunosuppressive TME is highly imperative.

**Results:**

In this article, we reported that Fe-doped and doxorubicin (DOX) loaded HA@Cu_2−X_S-PEG (PHCN) nanomaterials were rationally designed as targeted Fe-PHCN@DOX nano-nuclear-reactors, which evoked persistent T cell immune response together with anti-PD-L1 nanobodies. It was confirmed that nano-nuclear-reactors displayed strong nanocatalytic effect for effective antitumor effects. Consequently, they maximized the immunogenic cell death (ICD) effect for antigen presentation and then stimulated T cell activation. In addition, Fe-PHCN@DOX could reprogram M2-phenotype tumor-associated macrophages (TAMs) into M1-phenotype TAMs by relieving tumor hypoxia. Meanwhile, blockade of the anti-PD-L1 nanobody promoted T cell activation through targeting the PD-1/PD-L1 immunosuppressive pathway. Notably, in vivo tumor therapy verified that this nano-nuclear-reactor could be used as an excellent immunotherapy nanoplatform for tumor eradication and metastasis prevention with nanobody.

**Conclusions:**

Our findings demonstrated that nano-nuclear-reactors in combination with nanobody could evoke persistent T cell immune activation, suggesting them potential as a promising immunotherapy option for reversing immunosuppressive immune-cold tumors.

**Graphical Abstract:**

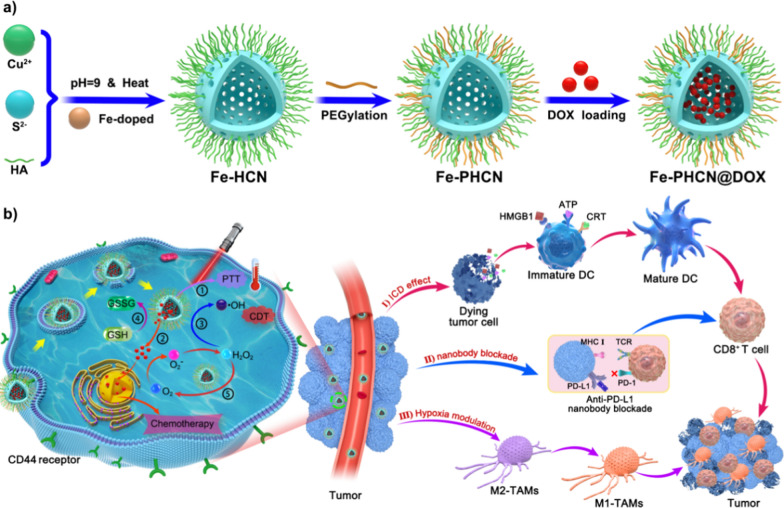

**Supplementary Information:**

The online version contains supplementary material available at 10.1186/s12951-022-01736-8.

## Background

In recent years, cancer immunotherapy has shifted the paradigm toward the treatment of cancers and is considered a promising strategy alternative to traditional anticancer drug [1–3]. Among them, immunotherapy based on T cell-mediated cellular immunity is attractive [4]. However, repressed T cells condition containing the absence of cytotoxic T cell infiltration and failure of T cell priming in the tumor microenvironment (TME) is a major challenge for succeeding antitumor immunotherapy [5, 6]. Low antigen presentation by dendritic cells (DCs), immunosuppression of tumor-associated macrophages (M2-type macrophages), and the inactivation of programmed death-1 (PD-1)/programmed death-ligand 1 (PD-L1) immunosuppressive pathway, mainly contribute to reduce the effectiveness of T cell-triggered immune responses [7–9]. Therefore, it is highly urgent to develop novel therapeutic approaches to simultaneously overcoming these obstacles that revitalized antitumor T cell immune responses.

Tumor immunogenic cell death (ICD) can release tumor-associated antigen (TAA) and damage-associated molecular patterns (DAMPs), which are beneficial to the recruitment and antigen presentation of DCs and then stimulating antitumor T cells [10–15]. Sufficient antigen presentation of DCs by the ICD inducers is the key to activating CD8^+^ T lymphocytes, thus determining the efficacy of antitumor immunotherapy [16, 17]. Additionally, the hypoxia tumor microenvironment generally impedes immune responses to tumors by promoting the polarization of immune-supportive M1-type tumor-associated macrophages (TAMs) into immunosuppressive M2-type TAMs [18, 19]. M2-type macrophages have been shown to induce T cells dysfunction and further reduce proliferation, then increase T cells apoptosis of antigen-specific CD8^+^ T cells by releasing cytokines, stromal proteins, and proteases [20, 21]. Reeducating TAMs to M1-type state by hypoxia alleviation can trigger robust T cell-mediated immune response for effective cancer immunotherapy. Moreover, the antitumor-specific T cell responses are also severely abolished by PD-1/PD-L1 immunosuppressive pathway [9, 22]. The overexpressed PD-L1 receptor on tumor cells impairs functioning T effector cells and inducing M2-type macrophages expansion and function, leading to the generation of the immunosuppressive tumor microenvironment [22]. The anti-PD-L1 monoclonal antibodies can effectively suppress the PD-1/PD-L1 pathway to increase the infiltration and function of T cell, thus amplifying effector T cells to eliminate the tumor [23–25]. Therefore, it is of great interest to create a nanobody-based therapy platform that can simultaneously overcome the three bottlenecks on-demand target sites for enhanced T cell-mediated immunotherapy efficacy.

Recently, photothermal therapy (PTT) can display high selectivity and low systemic toxicity to eradicate tumor cells by converting light energy into localized heat under near-infrared (NIR) laser irradiation. Our previous studies have confirmed that CuS-based nanomaterials (PHCN) could generate high PTT efficiency in the TME for the treatment of tumors [26]. Additionally, nanocatalytic therapy (NCT) as a non-invasive tumor-therapeutic modality has attracted increasing attention [27–30]. NCT agents can convert endogenous hydrogen peroxide (H_2_O_2_) to generate highly toxic hydroxyl radicals (•OH), which is a kind of reactive oxygen species (ROS), leading to induce irreversibly protein or DNA damage, and subsequently causing apoptosis and necrosis of tumor cells [31–33]. However, intracellular produced H_2_O_2_ levels are often insufficient to achieve satisfactory NCT efficacy [34, 35]. Thus, the development of NCT agents with H_2_O_2_-supplementing functionality and nanocatalytic enhancement is highly pursued.

**Fig. 1:**
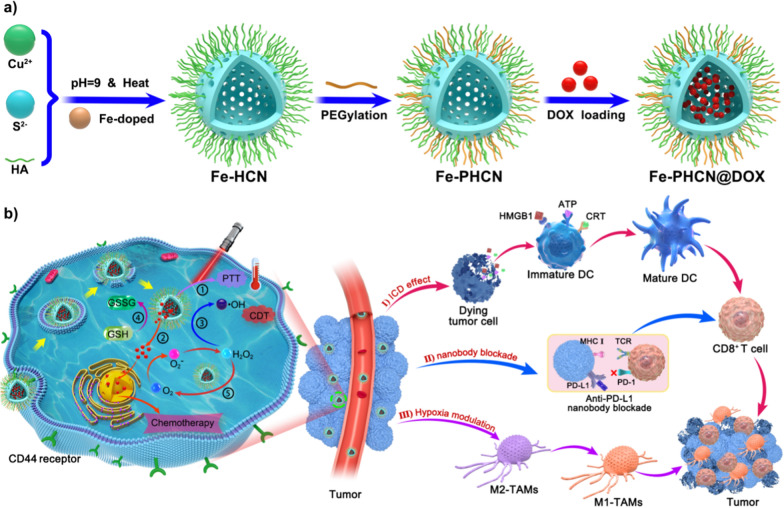
**a** Preparation procedure of the Fe-PHCN@DOX nano-nuclear-reactors. **b** Schematic illustration of synergistic combination of targeted nano-nuclear-reactors and anti-PD-L1 nanobodies evokes persistent T cell immune activation for cancer immunotherapy through (I) ICD effect; (II) nanobody blockade; (III) hypoxia modulation. The label ①,②,③,④,⑤ represents PTT effect, DOX-induced cytotoxicity and H_2_O_2_ generation, nanocatalytic effect, GSH depletion, catalase-like effect, respectively

In recent nanobodies have demonstrated significant translational potential in cancer therapy [36]. Nanobodies are the smallest functional antibodies which small size (~ 15 kDa) [37, 38]. In certain conditions, there are still defects in the use of monoclonal antibodies (mAbs) in cancer treatment. For example, mAbs with relatively large size result in limited penetration. Therefore, smaller antibody fragments, such as Fab, scFv, minibodies and nanobodies, have attracted attention. Nevertheless, nanobodies can overcome most of the above difficulties due to their better tissue permeability and high stability. So, the anti-PD-L1 nanobodies were selected to use in our study for better tumor treatment effect. In this work, we report a novel strategy of nano-nuclear-reactors that reverse low immunogenicity, hypoxia, and immunosuppressive immune-cold tumors to revitalize antitumor T cell immune responses in combination with the anti-PD-L1 nanobody. Hyaluronic acid (HA) not only can act as a targeting ligand to recognize abundant HA receptors (CD44) on specific tumor cells, but also serve as a capping stabilizer to synthesize Fe-HCNs. Cu and S can react to form the Cu_2−x_S nanomaterials, which display the photothermal and nanocatalytic effect, glutathione (GSH) peroxidase-like, and catalase-like activity. Doxorubicin (DOX) can act as a chemotherapy drug, and increase cellular H_2_O_2_ level in cancer cells via the redox cycles. Iron (Fe), a Fenton-like reaction agent, can transform H_2_O_2_ into hydroxyl radicals (•OH). Fe-doped and DOX loaded HA@Cu_2−x_S-PEG (PHCN) nanomaterials were rationally designed to fabricate the multifunctional nano-nuclear-reactors (denoted as Fe-PHCN@DOX). High NCT performance was achieved through the incorporation of Fe and DOX to PHCN nanomaterials by enhancing nanocatalytic efficiency and increasing the H_2_O_2_ level, respectively [39–41]. Additionally, the Fe-PHCN@DOX nano-nuclear-reactors could display glutathione (GSH) peroxidase-like activity for GSH depletion capacity and photothermal effect under near-infrared (NIR) light irradiation, further enhancing the NCT efficiency [42–44]. Moreover, the surface modification with hyaluronic acid (HA) and PEG was favorable for tumor-targeting ability and biocompatibility, respectively. Our multifunctional nano-nuclear-reactors combined with anti-PD-L1 nanobodies can effectively stimulate DCs maturation, polarize toward M1-type macrophage, and interference PD-1/PD-L1 pathway, thus triggering effective anti-tumor T cell immunity against primary and distant tumors, and tumor metastasis. This designed nano-nuclear-reactor combined with nanobody-mediated nanoplatform will achieve three functional applications (Fig. 1): (1) the Fe-PHCN@DOX nano-nuclear-reactors as the effective ICD amplifiers lead to high •OH generation, GSH depletion, photothermal effect, and DOX cytotoxicity; (2) Fe-PHCN@DOX nano-nuclear-reactors possess catalase-like activity that can efficiently catalyze H_2_O_2_ to produce O_2_, leading to alleviate tumor hypoxia for polarize M2-type macrophages into M1-type macrophages; (3) anti-PD-L1 nanobodies can be developed as novel PD-L1 immune checkpoint blockade alternative to the traditional monoclonal antibody [45]. Importantly, to the best of our knowledge, the use of targeted nano-nuclear-reactors together with anti-PD-L1 nanobodies to persistently amplify T cell immune responses for enhanced nanocatalytic-immunotherapy have not been previously reported.

## Results and discussion

### Preparation and characterization of Fe-PHCN@DOX nano-nuclear-reactors

**Fig. 2:**
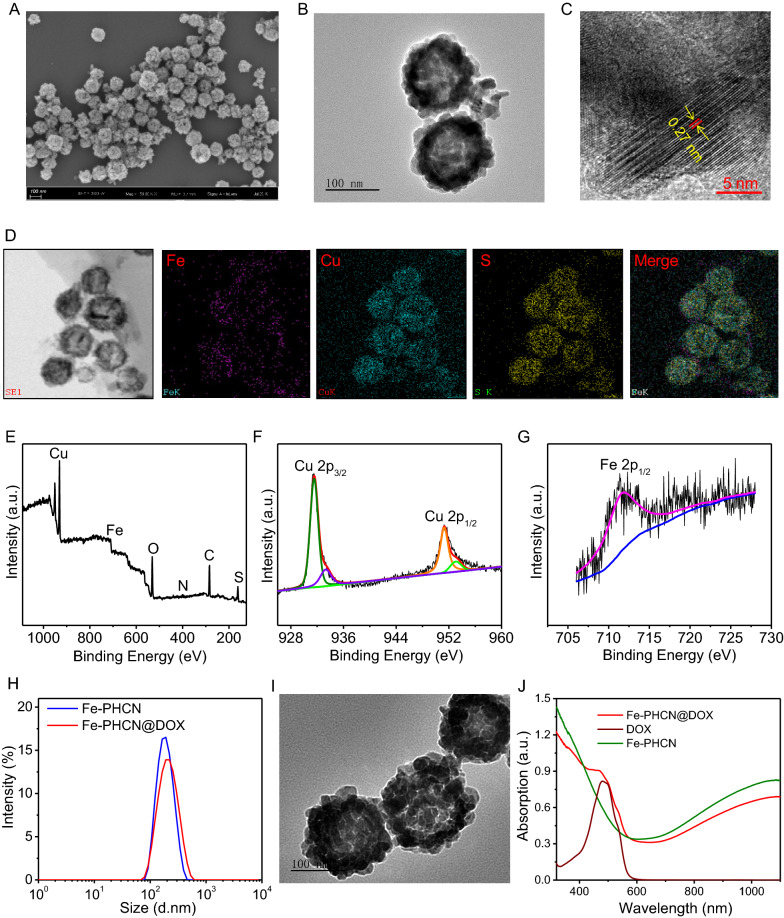
Characterization of the nano-nuclear-reactors. SEM (**A**) and TEM (**B**) image of Fe-PHCN. The scale bar represents 100 nm. **C** HRTEM images of Fe-PHCN. The scale bar represents 5 nm. **D** EDS elemental mapping of Fe-PHCN. **E** XPS characterization of Fe-PHCN. XPS high-resolution scans of Cu (**F**) and Fe (**G**) in Fe-PHCN. **H** Size of Fe-PHCN and Fe-PHCN@DOX by DLS measurement. **I** TEM image of Fe-PHCN@DOX. The scale bar represents 100 nm. **J** UV–vis spectrum of Fe-PHCN, DOX, and Fe-PHCN@DOX

Here, the Fe-PHCN nano-nuclear-reactors were facilely prepared as described in the Experiment Section by introduction of Fe^3+^ to PHCN nanomaterials (Fig. 1). Fe-PHCN nano-nuclear-reactors were used for effective loading DOX to construct the multifunctional Fe-PHCN@DOX nano-nuclear-reactors. Representative scanning electron microscopy (SEM) showed that the Fe-PHCN nano-nuclear-reactors exhibited excellent dispersion and monodisperse spherical shape (Fig. 2A; Additional file 1: Fig. S1). Transmission electron microscopy (TEM) images showed that Fe-PHCN nano-nuclear-reactors possessed hollow characteristics and uniform size approximately 130 nm (Fig. 2B; Additional file 1: Fig. S2). The nano-nuclear-reactors with a thickness shell of ≈ 20 nm exhibited an obvious hollow structure. The hydrodynamic diameter of Fe-PHCN nano-nuclear-reactors determined by dynamic light scattering (DLS) was approximately 191 nm (Additional file 1: Fig. S3), which was slightly higher than TEM average diameter due to the hydration effect. The lattice spacing (d-spacing) of the nano-nuclear-reactors was about 0.27 nm (Fig. 2C). The composition analysis of the nano-nuclear-reactors via TEM elemental mapping with Fe, Cu, and S elements was observed, suggesting Fe successfully doped to PHCN nanomaterials (Fig. 2D). X-ray photoelectron spectroscopy (XPS) and energy-dispersive X-ray spectroscopy (EDX) revealed that Fe, Cu, S, O, C, N were presented in the sample, further demonstrating the successful preparation of Fe-PHCN nano-nuclear-reactors (Fig. 2E; Additional file 1: Fig. S4). In the XPS data (Fig. 2F), the peaks located at 951.3/953.1 and 931.5/933.3 eV respectively correspond to Cu 2p1/2 and Cu 2p3/2 levels, confirming the coexistence of Cu^+^ and Cu^2+^. Meanwhile, the characteristic peaks of 711.5 eV accord with the Fe 2p1/2 (Fig. 2G), suggesting the existence of Fe^3+^ in nano-nuclear-reactors [46]. The zeta potential dropped from − 19.3 mV down to − 13.05 mV after PEGylation (Additional file 1: Fig. S5), demonstrating the successful modification of PEG. To further confirm the modification of PEG, we measured the FT-IR spectra of Fe-HCN and Fe-PHCN. FT-IR spectra clearly demonstrated that the specifically characteristic peaks for –C–O–C– were obviously enhanced after PEG modification (Additional file 1: Fig. S6), indicating the successful modification of PEG on nano-nuclear-reactors. The UV–vis absorption spectra showed the Fe-PHCN solutions possessed strong absorbance in the NIR I and II region (Additional file 1: Fig. S7).

DOX and the as-prepared Fe-PHCN were mixed to prepare Fe-PHCN@DOX nano-nuclear-reactors after stirring and centrifugation. Because of their uniquely hollow porous structure, the nano-nuclear-reactors could be used as a drug nanocarrier. Small DOX molecules (size of 1.53–1.19 nm) could act as guest molecules spread into the hollow interior in doses sufficient through the mesoporous shells. Additionally, positively charged DOX could bind the negatively charged Fe-PHCN to fabricate the Fe-PHCN@DOX via electrostatic interactions. As shown in Fig. 2H, the size of the Fe-PHCN@DOX was approximately 204 nm without an obvious increase in size relative to the original Fe-PHCN. TEM images showed that some morphologically inhomogeneous agglomerates were observed in the hollow interior of Fe-PHCN@DOX compared to that observed for the Fe-PHCN (Fig. 2I), which might be attributed to the DOX loaded into the hollow core. From UV–vis–NIR absorption spectra (Fig. 2J), Fe-PHCN@DOX exhibited new absorption peak at near 480 nm after the integration of the DOX. The results were also validated from an evident change of solution color before and after DOX loading (Additional file 1: Fig. S8). Therefore, these results demonstrated DOX successful loading into the nano-nuclear-reactors. The loading efficiency of the DOX in the nano-nuclear-reactors was approximately 36.67 wt%, as determined by the drug loading experiment. Additionally, the stability of the Fe-PHCN@DOX nano-nuclear-reactors in various solutions, including water, PBS, and DMEM medium containing 10% FBS, was further evaluated for 6 days through DLS measurement. It was observed that no obvious size changes of the Fe-PHCN@DOX in the various solutions (Additional file 1: Fig. S9), demonstrating the excellent stability of the nano-nuclear-reactors in water and physiological solution.

### Photothermal effect and pH/NIR responsible drug release

**Fig. 3:**
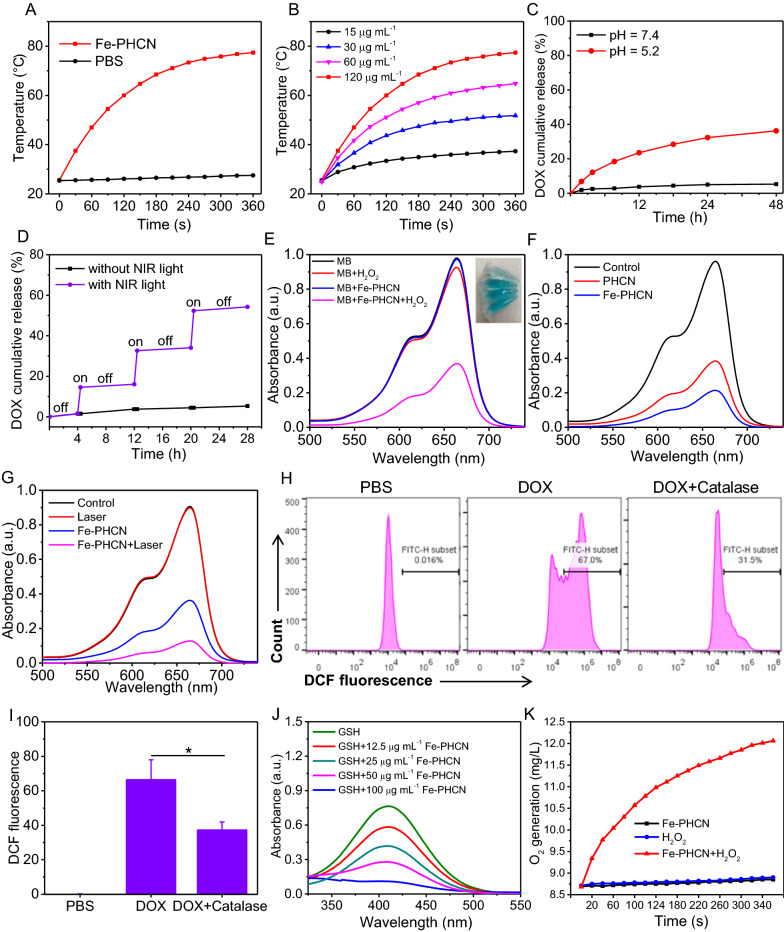
Multiple properties of Fe-PHCN@DOX nano-nuclear-reactors. **A** Temperature elevation curves of PBS and Fe-PHCN exposed to a 1064 nm NIR laser for 6 min. **B** Temperature elevation curves of Fe-PHCN with different concentrations under laser irradiation. Release curves of DOX from Fe-PHCN@DOX at different pH (**C**) and with or without NIR irradiation (**D**). **E** UV − vis spectra of MB aqueous solution from different groups. **F** UV − vis spectra of MB aqueous solution with Fe-PHCN and PHCN treatment. **G** UV − vis spectra of MB in Fe-PHCN solution treated with/without laser irradiation. **H**, **I** Intracellular ROS content was analyzed under DOX and DOX plus Catalase treatments using DCF as a ROS sensor. **J** GSH depletion under different concentrations of Fe-PHCN. (K) O_2_ generation of Fe-PHCN under with/without H_2_O_2_ treatment. The *p* values were analyzed using the Log-rank (Mantel-Cox) test. Data are presented as the mean ± standard error of the mean. *p < 0.05, **p < 0.01, ***p < 0.001

Based on the strong NIR absorption, the photothermal properties of the nano-nuclear-reactors were investigated by monitoring the temperature changes under NIR laser irradiation using an infrared thermal imaging camera. As shown in Fig. 3A, temperature of the Fe-PHCN solutions markedly increased under 1064 nm laser irradiation at 1 W cm^−2^ for 6 min, while no obvious temperature changes of PBS were observed. Moreover, the Fe-PHCN showed excellent concentration-dependent photothermal properties under laser irradiation (Fig. 3B). These results showed that the excellent photothermal efficiency of the Fe-PHCN could allow it to be an efficient photothermal agent. DOX release behaviors from Fe-PHCN@DOX at different pH values (7.4 and 5.2) were investigated. Fe-PHCN@DOX displayed sustained drug release behavior (Fig. 3C), and the amount of DOX released increased with decreasing pH due to reduced electrostatic interactions between the DOX and the Fe-PHCN in acidic pH conditions. As shown in Fig. 3D, only a small amount of DOX was observed without NIR light, while a burst release of DOX occurred under NIR light irradiation. The further increased DOX release under repeated laser irradiation was attributed to the photothermal effect of the Fe-PHCN, thus generating heat to promote drug diffusion.

### Nanocatalytic activity, GSH depletion, and O_2_ generation of Fe-PHCN nano-nuclear-reactors

To further investigate nanocatalytic properties, the •OH generation of Fe-PHCN was determined using the methylene blue (MB) degradation method [47]. No significant changes of MB absorption were observed in H_2_O_2_ or Fe-PHCN compared to Fe-PHCN plus H_2_O_2_ (Fig. 3E), indicating that the Fe-PHCN could catalyze H_2_O_2_ to generate •OH. The nanocatalytic properties of Fe-PHCN in a concentration-dependent manner were confirmed (Additional file 1: Fig. S10). As shown in Fig. 3F, higher •OH generation by Fe-PHCN was found compared to PHCN, suggesting that the introduction of Fe^3+^ enhancing nanocatalytic efficiency [39]. Fe-PHCN under the 1064 nm laser irradiation could increase •OH generation (Fig. 3G), which revealed that photothermal effect mediated high temperature could improve the nanocatalytic effect [48, 49]. Additionally, DOX contributing to producing H_2_O_2_ in cancer cells was evaluated using the sensor 2ʹ,7ʹ-dichlorofluorescein diacetate (DCF) [50]. As shown in Fig. 3H, I, high green fluorescence was observed in cells after DOX treatment, suggesting high H_2_O_2_ level. In contrast, the cells treated with Catalase, a H_2_O_2_ scavenger, showed relatively low ROS level. These results demonstrated that DOX can generate H_2_O_2_ in cancer cells, potentially enhancing nanocatalytic efficiency. Additionally, the GSH depletion properties of Fe-PHCN were investigated using the GSH kit. As shown in Fig. 3J, the content of GSH was gradually decreased with the increasing concentrations of Fe-PHCN. The results revealed that Fe-PHCN exhibited excellent GSH depletion ability. The catalase-like activity of Fe-PHCN was investigated by monitoring O_2_ production using a dissolved oxygen meter. Higher O_2_ generation was observed treated with Fe-PHCN plus H_2_O_2_, while no obvious O_2_ production treated with Fe-PHCN were observed (Fig. 3K), which demonstrated that the Fe-PHCN could effectively generate O_2_ to relieve tumor hypoxia.

### Cellular uptake and therapeutic efficacy

**Fig. 4:**
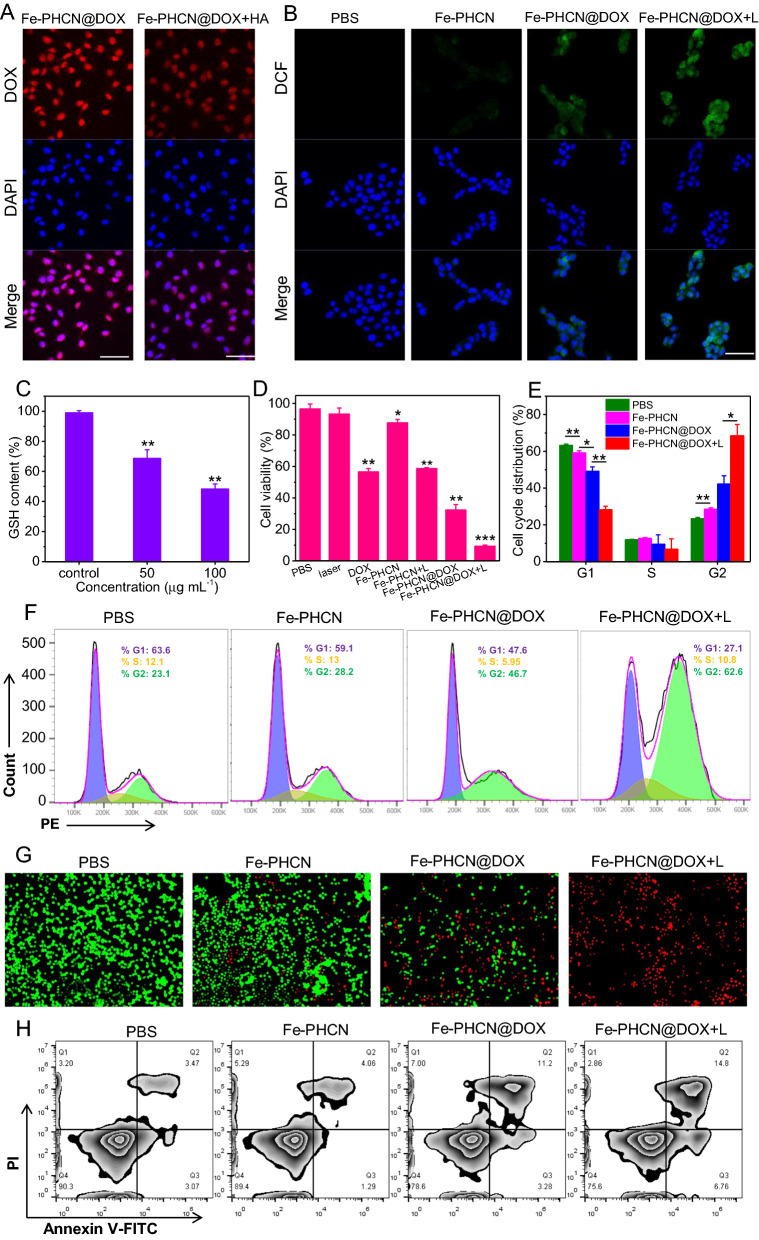
Cellular uptake and therapeutic efficacy of Fe-PHCN@DOX nano-nuclear-reactors. **A** Confocal images of the CT26 subcellular localization of the Fe-PHCN@DOX with/without HA treatment. The scale bar represents 50 μm. **B** Fluorescence images of DCF-stained CT26 cells under different group treatment. The scale bar represents 50 μm. **C** Intracellular GSH depletion under different concentrations of Fe-PHCN@DOX. **D** Cell survival rate after different group treatment by the CCK-8 assay. **E**, **F** Cell cycle arrest in G2 phase was measured under different group treatment. **G** Fluorescence images of calcein AM (green, live cells) and PI (red, dead cells) costained CT26 cells after different group treatment. The scale bar represents 100 μm. **H** Flow cytometry analysis of apoptosis in CT26 cells treated with different groups. The *p* values were analyzed using the Log-rank (Mantel-Cox) test. Data are presented as the mean ± standard error of the mean. *p < 0.05, **p < 0.01, ***p < 0.001

The intracellular distribution and uptake of the Fe-PHCN@DOX nano-nuclear-reactors were evaluated by confocal microscopy and flow cytometry, respectively. As shown in Fig. 4A, the presence of free HA could significantly inhibit the intracellular distribution of the Fe-PHCN@DOX because of the competitive interaction between Fe-PHCN@DOX and HA on CD44 receptors on CT26 cells [51]. The flow cytometric results were consistent with those from confocal microscopy (Additional file 1: Fig. S11), suggesting the HA-mediated targeting property of the Fe-PHCN@DOX. To study cellular ROS production of the Fe-PHCN@DOX nano-nuclear-reactors, a fluorescent probe DCF was used. Highest green fluorescent intensity was observed in cells treated with Fe-PHCN@DOX plus laser irradiation compared to other groups (Fig. 4B; Additional file 1: Fig. S12), suggesting that Fe-PHCN@DOX could generate more •OH in cells by introducing Fe^3+^/DOX and using laser irradiation. These results confirmed that the Fe-PHCN@DOX nano-nuclear-reactors displayed superior nanocatalytic efficacy in cancer cells. Meanwhile, the dramatic depletion of intracellular GSH was detected in cancer cells treated with the Fe-PHCN@DOX nano-nuclear-reactors (Fig. 4C). This finding revealed that Fe-PHCN@DOX exhibited excellent intracellular GSH depletion ability, potentially enhancing the nanocatalytic effect by destroying cellular antioxidant defense system. The therapeutic efficacy of the Fe-PHCN@DOX nano-nuclear-reactors was evaluated by a CCK-8 assay. As shown in Fig. 4D, the Fe-PHCN@DOX under laser irradiation induced a remarkably higher cell toxicity of 90.54%, far higher than that of other groups. This result revealed that Fe-PHCN@DOX could display optimal anticancer effect through photothermal effect, •OH generation, GSH depletion, and DOX-induced cytotoxicity. Additionally, cell cycle progression was analyzed using PI staining. The higher proportion of cells treated with Fe-PHCN@DOX plus laser (62.6%) in the G2 phase was observed than that of PBS (23.1%), Fe-PHCN (28.2%), and Fe-PHCN@DOX (46.7%) (Fig. 4E, F), which suggested that the Fe-PHCN@DOX nano-nuclear-reactors could induce cell cycle arrest in the G2 phase. Moreover, fluorescence staining of the living/dead cells also showed that the Fe-PHCN@DOX nano-nuclear-reactors under laser irradiation caused more cell death as compared with that in other groups (Fig. 4G). Annexin V-FITC/PI cell co-staining (Fig. 4H; Additional file 1: Fig. S13) further confirmed that the Fe-PHCN@DOX nano-nuclear-reactors displayed the effective anticancer efficacy under laser irradiation.

### Nanobody binding activity, cellular O_2_ production, and ICD induction

**Fig. 5:**
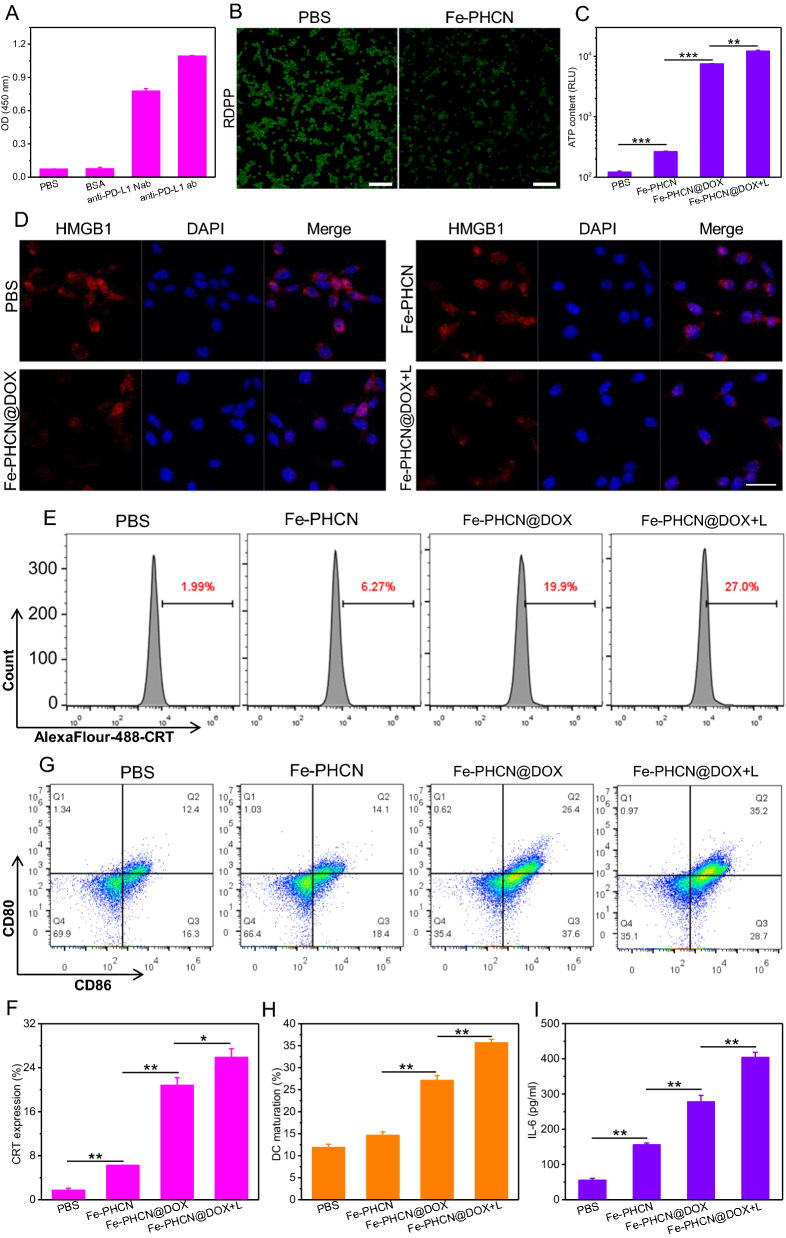
Nanobody binding activity, cellular O_2_ production, and ICD induction of Fe-PHCN@DOX nano-nuclear-reactors. **A** The binding activity of PD-L1 antigen with BSA, anti-PD-L1 nanobody (Nab), and anti-PD-L1 antibody (ab) was measured by ELISA. **B** O_2_ generation in CT26 cells treated with different groups. The scale bar is 100 μm. The ATP content (**C**), the HMGB1 level in nuclei (**D**), and the CRT expression on the surface of CT26 cells (**E**, **F**) after different group treatment. **G**, **H** Quantification of CD80 and CD86 expression on the surface of DC2.4 cells after different treatment by flow cytometry. **I** ELISA analysis of the levels of cytokines IL-6 secreted by DC2.4 cells in the medium. The scale bar is 25 μm. The *p* values were analyzed using the Log-rank (Mantel-Cox) test. Data are presented as the mean ± standard error of the mean. *p < 0.05, **p < 0.01, ***p < 0.001

To verify our hypothesis that the nano-nuclear-reactors induced effective immune activation of T cells by reversing immunosuppression in low immunogenicity and hypoxia tumor, we detect the PD-L1/anti-PD-L1 nanobody binding activity, the O_2_ production, and ICD effect in vitro. The molecular weight of anti-PD-L1 nanobody was significantly lower than that of the anti-PD-L1 antibody in protein gel electrophoresis (Additional file 1: Fig. S14), confirming the successful preparation of the nanobody. Anti-PD-L1 nanobody exhibited stronger tissue permeability and distribution in tumor tissue than that of commercial anti-PD-L1 monoclonal antibody (Additional file 1: Fig. S15). Figure 5A showed that the anti-PD-L1 nanobody/PD-L1 antigen binding activity was similar to the commercial antibody. Moreover, the anti-PD-L1 nanobody could effectively bind the PD-L1 receptor on CT26 cells (Additional file 1: Fig. S16). These results indicated that the anti-PD-L1 nanobody can blockade the PD-1/PD-L1 immunosuppressive pathway. The levels of TNF-α and IFN-γ released from T cells were obviously increased under anti-PD-L1 nanobody treatment (Additional file 1: Fig. S17), suggesting nanobody-mediated PD-L1 blockade promoting T cell activation. We further measured PD-L1 blockade-mediated T cell activation by staining the activation maker CD69. The percentage of CD69^+^CD8^+^ T cells was significantly increased under anti-PD-L1 nanobody treatment (Additional file 1: Fig. S18), suggesting nanobody-mediated PD-L1 blockade promoting T cell activation. The cellular O_2_-generating activities of Fe-PHCN using the O_2_ probe [Ru(dpp)_3_]Cl_2_ (RDPP). As shown in Fig. 5B, compared to the PBS group, lower green fluorescence of RDPP was observed in cells treated with Fe-PHCN. Additionally, the increased M1-type macrophages (CD11b^+^F4/80^+^CD86^+^CD206^−^) and decreased M2-type macrophages (CD11b^+^F4/80^+^CD86^−^CD206^+^) were observed under Fe-PHCN treatment (Additional file 1: Fig. S19). The results demonstrated that the Fe-PHCN could effectively produce O_2_, thus potentially polarizing M2-type macrophages into M1-type macrophages. To evaluate the effect of the Fe-PHCN@DOX nano-nuclear-reactors-induced ICD, we selected calcium netting protein (CRT), adenosine triphosphate (ATP), and high mobility group box 1 (HMGB1) as indicators [52, 53]. Compared with PBS group, the increased ATP and CRT level, and decreased HMGB1 level in nuclei with Fe-PHCN and Fe-PHCN@DOX treatments was observed (Fig. 5C–F). In contrast, the group treated with Fe-PHCN@DOX plus laser irradiation induced significantly increased ATP secretion, CRT expression, and HMGB1 release from nuclei, suggesting that nano-nuclear-reactors could serve as an excellent ICD amplifier. The DAMPs released from CT26 tumor cells undergoing ICD were presented to immatured DCs for promoting DC maturation (CD11c^+^CD86^+^CD80^+^), which is responsible for activating T cell immune responses. DC2.4 cells were chosen for testing in vitro DCs maturation. The CT26 cells were pre-treated with nano-nuclear-reactors and then co-cultured immature DC2.4 cells. The frequency of matured DC2.4 cells was then analyzed by flow cytometry. Compared with PBS group (12.4%), the moderately increased DCs maturation (CD11c^+^CD86^+^CD80^+^) with Fe-PHCN (14.1%) and Fe-PHCN@DOX (26.4%) treatment was observed (Fig. 5G, H; Additional file 1: Fig. S20). Remarkably, the Fe-PHCN@DOX plus laser irradiation significantly promoted DCs maturation (35.2%). These results demonstrated that the Fe-PHCN@DOX nano-nuclear-reactors based ICD effect efficiently promoted DCs maturation for evoking T cell immune activation. Moreover, the secretion levels of pro-inflammatory cytokines IL-6 and TNF-α were investigated using enzyme-linked immunosorbent assay (ELISA). Compared with other groups, the secretion of IL-6 and TNF-α observed increased under Fe-PHCN@DOX treatment (Fig. 5I; Additional file 1: Fig. S21), which further promoted T cell activation.

### In vivo biodistribution and tumor microenvironment alterations by nano-nuclear-reactors

**Fig. 6:**
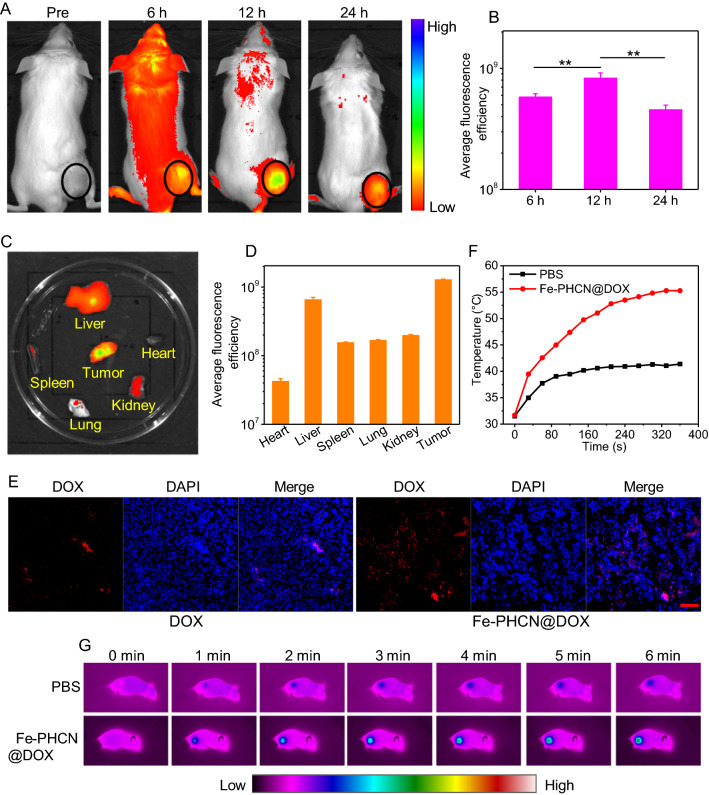
In vivo biodistribution and tumor microenvironment alterations by nano-nuclear-reactors. **A** In vivo fluorescence imaging of CT26 tumor-bearing mice intravenously injected with Fe-PHCN@DOX/Ce6 recorded under different time points. **B** The mean fluorescence intensity at the tumor sites was quantified using CRi maestro in vivo imaging system. Ex vivo fluorescence images (**C**) and the mean fluorescence intensity (**D**) of major organs and tumor dissected from mice injected with Fe-PHCN@DOX/Ce6. **E** The fluorescent signal of free DOX and Fe-PHCN@DOX in tumor tissues was recorded using confocal microscopy. The scale bar represents 50 μm. Temperature elevation curves (**F**) and infrared photothermal images (**G**) of tumor-bearing mice measured after intravenous injection of PBS and Fe-PHCN@DOX plus laser irradiation for 6 min. The *p* values were analyzed using the Log-rank (Mantel-Cox) test. Data are presented as the mean ± standard error of the mean. *p < 0.05, **p < 0.01, ***p < 0.001

High accumulation of nano-nuclear-reactors in tumors is a prerequisite for achieving enhanced antitumor therapeutic outcomes and lower side effect to tissues. To visualize the dynamic distribution of nano-nuclear-reactors in vivo, a NIR dye Chlorin e6 (Ce6) was incorporated into Fe-PHCN@DOX to generate Ce6-loaded Fe-PHCN@DOX (Fe-PHCN@DOX/Ce6) [50]. Tumor-bearing mice were intravenously injected with Fe-PHCN@DOX/Ce6, and the biodistribution was monitored at different time intervals using an in vivo imaging system. The fluorescence signals of Fe-PHCN@DOX/Ce6 in the tumor region increased with increasing time and reached a peak at 12 h after injection, and gradually decreased at 24 h (Fig. 6A, B). This finding revealed that the Fe-PHCN@DOX/Ce6 could efficiently enrich in the tumor region for a prolonged time. Moreover, direct ex vivo fluorescence imaging of excised tumors obtained at 24 h (Fig. 6C, D), confirmed the effective distribution of Fe-PHCN@DOX/Ce6 in tumor tissue, suggesting that HA targeting property significantly enhanced the tumor accumulation of the nano-nuclear-reactors. Meanwhile, the accumulation of DOX in tumors was also investigated after Fe-PHCN@DOX injection. The confocal images showed that Fe-PHCN@DOX significantly enhanced the fluorescent signals of DOX in tumors as compared with free DOX, which further confirmed effectively enhanced DOX accumulation in tumor tissues by Fe-PHCN@DOX nano-nuclear-reactors (Fig. 6E). Moreover, the dramatic decrease of GSH in tumor was observed with the increasing concentration of Fe-PHCN@DOX (Additional file 1: Fig. S22), which indicated that the nano-nuclear-reactors could regulate the TME. Additionally, to monitor thermal dynamics in tumor treated with the Fe-PHCN@DOX nano-nuclear-reactors after *i.v.* injection, photothermal imaging could be used to test the temperature variation of tumor by an infrared thermal camera. As shown in Fig. 6F, G, the temperature of the Fe-PHCN@DOX in the tumor region gradually increased with increasing laser irradiation time, and the temperature significantly increased to 55.27 °C within 6 min. In contrast, the temperature of the tumor treated with PBS was not remarkably elevated. Therefore, this result showed that the Fe-PHCN@DOX nano-nuclear-reactors could act as an excellent photothermal agent in vivo.

### Enhanced therapeutic efficacy on CT26 tumor in vivo

**Fig. 7:**
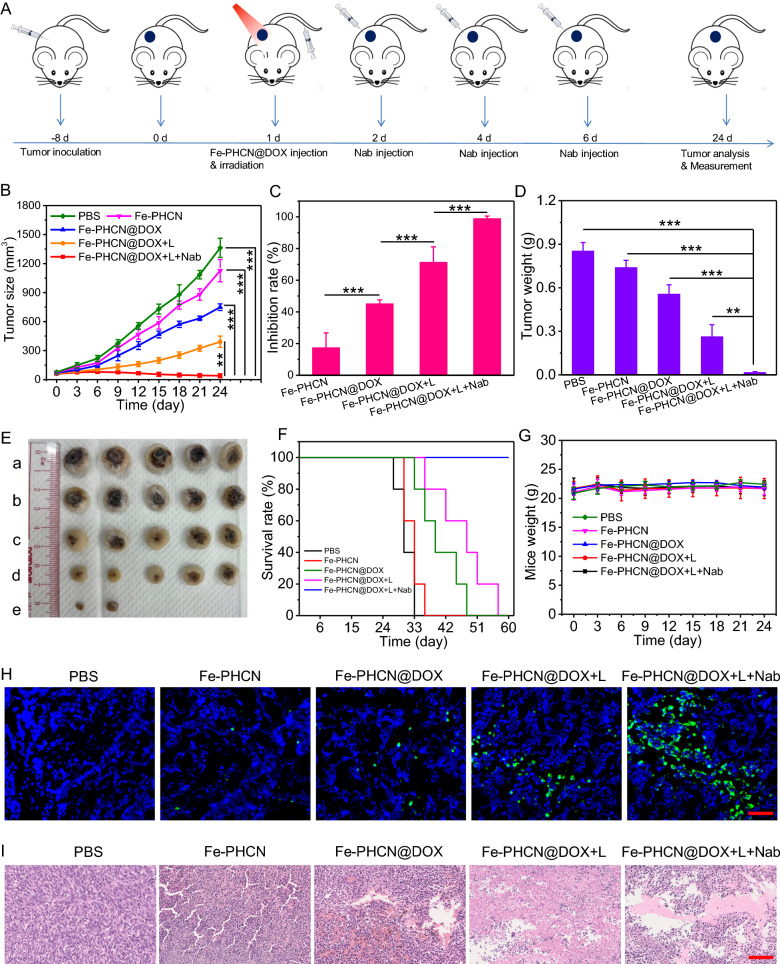
Antitumor therapeutic efficacy of on CT26 tumor in vivo. **A** Treatment schedule for Fe-PHCN@DOX and anti-PD-L1 nanobody (Nab) combination therapy. Tumor growth curves (**B**) and inhibition rates (**C**) from various groups, including a) PBS; b) Fe-PHCN; c) Fe-PHCN@DOX; d) Fe-PHCN@DOX plus laser; and e) Fe-PHCN@DOX plus laser plus anti-PD-L1 nanobody. Tumor weights (**D**) and representative photos of tumor size (**E**) of mice in each group. Survival rates (**F**) and body weights (**G**) of mice after different treatments. **H** Fluorescence images of TUNEL-stained tumor slices after various treatments. Cell nuclei were stained with DAPI (blue fluorescence). Green fluorescence indicates TUNEL-positive cells. **I** H&E-stained images of tumor slices obtained from different groups of mice. The scale bar represents 100 μm. The *p* values were analyzed using the Log-rank (Mantel-Cox) test. Data are presented as the mean ± standard error of the mean. *p < 0.05, **p < 0.01, ***p < 0.001

Motivated by the effective tumor accumulation and excellent photothermal performance in tumor of the Fe-PHCN@DOX nano-nuclear-reactors, we next evaluated the antitumor therapeutic potential. The xenograft CT26 tumor models were established, and tumor-bearing mice were randomly divided into five groups: (a) PBS; (b) Fe-PHCN; (c) Fe-PHCN@DOX; (d) Fe-PHCN@DOX plus laser irradiation; and (e) Fe-PHCN@DOX plus laser irradiation plus anti-PD-L1 nanobody. As shown in Fig. 7A, after 8 days’ tumor inoculation, the tumor volume reach about 60 mm^3^, and then tumor-bearing mice were intravenously injected with nano-nuclear-reactors on day 1. Then, tumors were performed under 1064 nm laser irradiation 12 h post-injection, and anti-PD-L1 nanobody was intra-tumorally injected on day 2, 4, and 6. Although systemic treatment administration of immune checkpoint inhibitors is conventional approaches, their serum pharmacokinetics is unpredictable. Particularly, antibody penetration from the circulation into solid tumors is limited. Meanwhile, systemic distribution may raise safety issues. Systemic inflammation often prevents the use of efficacious doses of immune checkpoint inhibitors [54]. Intratumoral delivery is an attractive option to increase the bioavailability of nanobody in TME [55, 56]. In the preliminary in vivo experiment, the Fe-PHCN@DOX displayed higher inhibition effect of tumor growth than that of PBS and free DOX (Additional file 1: Fig. S23). Tumor volumes were monitored every 3 days. Compared with the PBS group, the Fe-PHCN (17.3%) and Fe-PHCN@DOX (45.05%) groups exhibited the partly inhibition effect of tumor growth (Fig. 7B). In contrast, the Fe-PHCN@DOX group could induce the higher tumor suppression effect (71.29%) plus laser irradiation treatment. Moreover, the group of Fe-PHCN@DOX plus laser plus anti-PD-L1 nanobody resulted in a strongest tumor suppressing effect (98.82%), which attributable to the enhanced antitumor therapeutic effect (Fig. 7C). Meanwhile, the group of Fe-PHCN@DOX plus laser plus anti-PD-L1 nanobody could effectively reduce tumor weight (Fig. 7D), almost ablate the tumor size (Fig. 7E), and significantly prolonged the survival rates of the mice (Fig. 7F) as compared with other groups. No significant variations in mice weight were observed in any of the groups during the treatment process, indicating the excellent biosafety of the Fe-PHCN@DOX nano-nuclear-reactors (Fig. 7G). Furthermore, to evaluate the antitumor therapeutic outcome, hematoxylin and eosin (H&E) and terminal deoxynucleotidyl transferase dUTP nick-end labeling (TUNEL) staining was used to analyze the tumors. Compared with other groups, Fe-PHCN@DOX under laser irradiation together with anti-PD-L1 nanobody could induce remarkable tumor tissue damage (Fig. 7H) and more apoptosis/necrosis of tumors (Fig. 7I). Taken together, these results demonstrated that the Fe-PHCN@DOX nano-nuclear-reactors plus PD-L1 immune checkpoint blockade could effectively improve the antitumor therapeutic effectiveness.

### In vivo ICD induction and immunosuppressive TME reprogramming

**Fig. 8:**
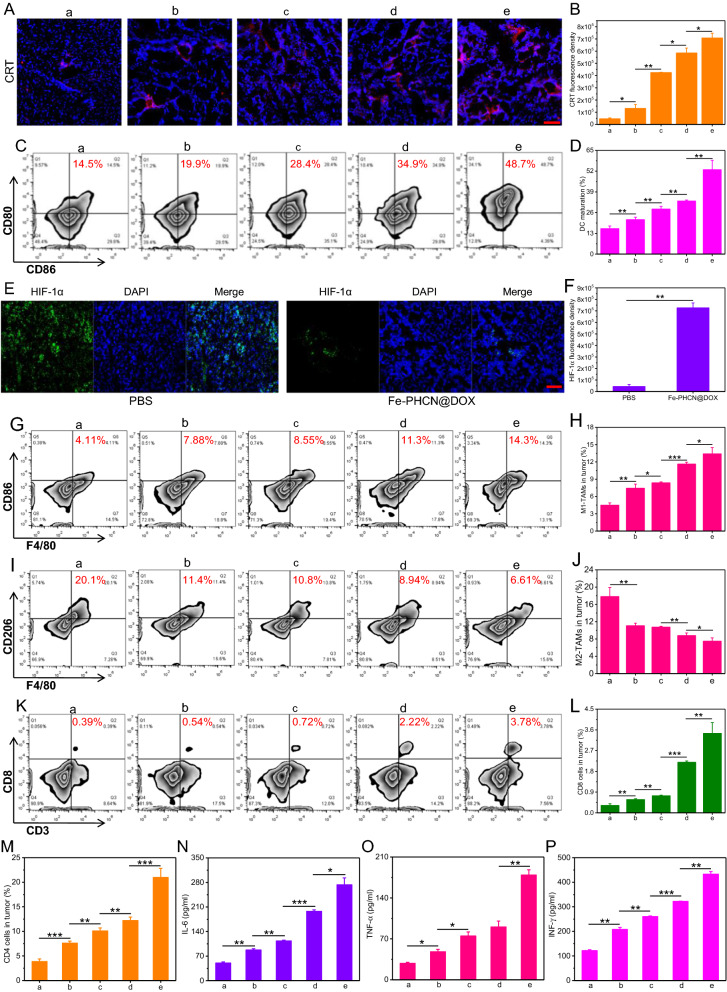
In vivo immunogenic cell death induction and immunosuppressive TME reprogramming. Confocal microscopy images (**A**) and fluorescence intensity (**B**) of CRT protein exposure in tumor tissues treated with a) PBS; b) Fe-PHCN; c) Fe-PHCN@DOX; d) Fe-PHCN@DOX plus laser; and e) Fe-PHCN@DOX plus laser plus anti-PD-L1 nanobody. The quantification (**C**) and percentage (**D**) of matured DCs cells (CD11c^+^CD86^+^CD80^+^) by flow cytometric analyses after various treatments. Representative immunofluorescence images (**E**) and relative quantitative analysis (**F**) of hypoxia areas in tumors treated with different groups after staining with DAPI (blue) and anti-HIF-1α antibody (green). The quantification (**G**) and percentage (**H**) of M1-type macrophages (CD11b^+^F4/80^+^CD86^+^) by flow cytometric analyses after various treatments. The quantification (**I**) and percentage (**J**) of M2-type macrophages (CD11b^+^F4/80^+^CD206^+^) by flow cytometric analyses after various treatments. The quantification (**K**) and percentage (**L**) of CD8^+^ cytotoxic T (CD3^+^CD8^+^) cells by flow cytometric analyses after various treatments. **M** The percentage of CD4^+^ helper T (CD3^+^CD4^+^) cells by flow cytometric analyses after various treatments. **N**–**P** ELISA analysis of the levels of cytokines IL-6, TNF-⍺, and IFN-γ in serum of mice after various treatments. The scale bar represents 50 μm. The *p* values were analyzed using the Log-rank (Mantel-Cox) test. Data are presented as the mean ± standard error of the mean. *p < 0.05, **p < 0.01, ***p < 0.001

On the basis of the excellent antitumor efficacy, the potential mechanism that nano-nuclear-reactors and nanobody mediated persistently amplifying T cell immune response was explored in vivo. Firstly, the ICD effect in tumors and DCs maturation in lymph nodes were investigated. As shown in Fig. 8A, B, remarkably higher CRT expression was observed in tumor tissue treated with Fe-PHCN@DOX plus laser plus nanobody group as compared with other groups, suggesting their superior capacity in inducing potent ICD effect for the in vivo DCs maturation. As expected, the Fe-PHCN@DOX plus laser plus nanobody group could effectively promote DCs maturation (CD11c^+^CD86^+^CD80^+^) with higher percentage of matured DCs (48.7%) than that of PBS (14.5%), Fe-PHCN (19.9%), Fe-PHCN@DOX (28.4%), and Fe-PHCN@DOX plus laser (34.9%) groups (Fig. 8C, D; Additional file 1: Fig. S24). Therefore, after tumor apoptosis induced by the ICD effect, the released DAMPs could be presented to immatured DCs to effectively simulate DCs maturation, thereby potentially stimulating T cell activation. Secondly, the hypoxic condition and macrophages polarization in TME were analyzed. From immunofluorescent staining images, the high green fluorescence signals of hypoxia inducible factors-1 alpha (HIF-1α) in tumors treated with PBS was observed, while the lower green fluorescence signals of HIF-1α in tumors treated with the Fe-PHCN@DOX (Fig. 8E, F). This finding revealed that Fe-PHCN@DOX nano-nuclear-reactors can effectively relieve hypoxia in tumors, potentially polarizing toward M1-type macrophage. More M1-type macrophage (CD11b^+^F4/80^+^CD86^+^) percentage (14.3%) in tumors of mice treated with the Fe-PHCN@DOX plus laser plus nanobody group was observed as compared with PBS (4.11%), Fe-PHCN (7.88%), Fe-PHCN@DOX (8.55%), and Fe-PHCN@DOX plus laser (11.3%) groups (Fig. 8G, H; Additional file 1: Fig. S25A). The Fe-PHCN@DOX plus laser plus nanobody group also effectively decreased the percentage of M2-type macrophages (CD11b^+^F4/80^+^CD206^+^) in tumors (Fig. 8I, J; Additional file 1: Fig. S25B). These results showed strong evidence that the polarization of M2-type macrophages to M1-type macrophages by relieving tumor hypoxia, thus potentially enhancing T cell immune function. Finally, T cell-mediated cellular immunity including the activation and infiltration of CD8^+^ cytotoxic T (CD3^+^CD8^+^) cells and CD4^+^ helper T (CD3^+^CD4^+^) cells in TME was investigated. The highest percentage of CD8^+^ cytotoxic T cells (3.78%) in tumors in Fe-PHCN@DOX plus laser plus nanobody group was found when compared with PBS (0.39%), Fe-PHCN (0.54%), Fe-PHCN@DOX (0.72%), and Fe-PHCN@DOX plus laser (2.22%) groups (Fig. 8K, L; Additional file 1: Fig. S26A). Consistent with CD8^+^ cytotoxic T cells, the Fe-PHCN@DOX plus laser plus nanobody group also remarkably increased the highest percentage of CD4^+^ helper T cells (Fig. 8M; Additional file 1: Fig. S26B, S27). These results demonstrated the triggering effective T cell activation and infiltration in TME by ICD effect, macrophages polarization, and nanobody blockade. To further study the anti-tumor mechanism of CD8^+^ and CD4^+^ T cells, we determined the proportion of IFN-γ^+^, Granzyme B^+^ and Perforin^+^ in CD8^+^ T cells as well as IFN-γ^+^ and TNF-α^+^ in CD4^+^ cells isolated from tumor tissues. The group of Fe-PHCN@DOX plus laser plus anti-PD-L1 nanobody could trigger higher percentage of IFN-γ^+^, Granzyme B^+^, Perforin^+^ CD8^+^ T cells (Additional file 1: Fig. S28), and IFN-γ^+^, TNF-α^+^ CD4^+^ cells (Additional file 1: Fig. S29) in tumor tissue than that of other group. Compared to other treatment, the lower percentage of Treg cells (CD3^+^CD4^+^Foxp3^+^) in tumor tissue was observed after the Fe-PHCN@DOX plus laser plus nanobody treatment (Additional file 1: Fig. S30). Importantly, Fe-PHCN@DOX plus laser plus nanobody showed a higher number of infiltrating T cells in tumors than that of other group (Additional file 1: Fig. S31). The above results indicated that nano-nuclear-reactors combined with nanobody could effectively activate and strengthen CD8^+^ cytotoxic T cells and CD4^+^ helper T cells, thus significantly enhancing the anti-tumor efficacy. Additionally, the levels of cytokines including IL-6, TNF-α, and interferon-γ (IFN-γ) in the blood were evaluated. The higher levels of these cytokines were found after the Fe-PHCN@DOX plus laser plus nanobody treatment than those of other groups (Fig. 8N–P). Collectively, the above results indicated that nano-nuclear-reactors together with nanobody promoted the ICD-mediated DCs maturation, macrophages polarization, and anti-PD-L1 blockade for effective antitumor T cell immune response by reversing the low immunogenicity, hypoxia, and immunosuppressive TME.

### Abscopal effect and metastasis prevention

**Fig. 9:**
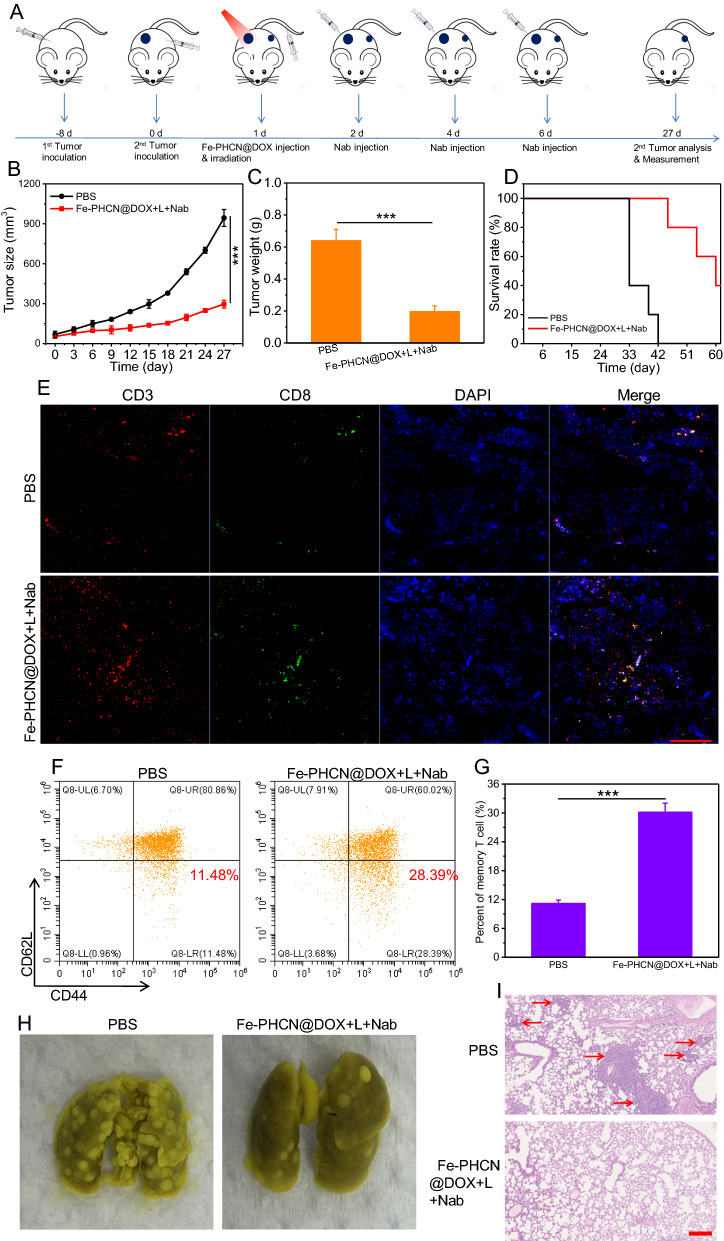
Abscopal effect and lung metastasis prevention in vivo. **A** Treatment schedule for Fe-PHCN@DOX and anti-PD-L1 nanobody combination therapy. Tumor growth curves (**B**) and tumor weights (**C**) from different groups. **D** Survival rates of mice after different treatments. **E** CLSM images of CD3^+^CD8^+^ T cells after staining with DAPI (blue), anti-CD3 antibody (red) and anti-CD8 antibody (green), respectively. The scale bar is 100 µm. The quantification (**F**) and percentage (**G**) of the effector memory T cells (CD3^+^CD8^+^CD44^+^CD62L^−^) by flow cytometric analyses after different treatments. **H** Representative images of the lung metastatic nodules. **I** H&E staining of lungs after different treatments. The scale bar is 200 μm. The *p* values were analyzed using the Log-rank (Mantel-Cox) test. Data are presented as the mean ± standard error of the mean. *p < 0.05, **p < 0.01, ***p < 0.001

An important feature of immune systems is the ability of immune memory that protects organisms from tumor cells re-attacking and tumor metastasis. To further evaluate the immune memory effects of nano-nuclear-reactors together with nanobody, CT26 cells were seeded on the left/right side of the same mice on day − 8 and 0 (Fig. 9A). After the tumor volume reach about 60 mm^3^, and tumor-bearing mice were randomly divided into two groups: a) PBS; b) Fe-PHCN@DOX plus laser plus anti-PD-L1 nanobody. Then, tumor-bearing mice were intravenously injected with nano-nuclear-reactors on day 1. Then, the primary tumors were performed under 1064 nm laser irradiation 12 h post-injection, and anti-PD-L1 nanobody was intratumorally injected on day 2, 4, and 6. For primary tumors, almost complete elimination of the tumors with Fe-PHCN@DOX plus laser plus nanobody treatment was found, consistent with the above results. For distant tumors, the tumors volume was monitored by caliper measurement. As showed in Fig. 9B, the growth rates of distant tumors treated with PBS group were rapidly, while significant growth inhibition rates of distant tumors treated with Fe-PHCN@DOX plus laser plus nanobody group were observed. This finding indicated that nano-nuclear-reactors together with nanobody mediated nano-immunotherapy could trigger strong immune responses. Moreover, the Fe-PHCN@DOX plus laser plus nanobody group could effectively reduce the tumor weight and prolong the mice survival rates as compared with the PBS group (Fig. 9C, D). From immunofluorescent staining images, a significant increase of infiltrating CD8^+^ T cell in distant tumors in Fe-PHCN@DOX plus laser plus nanobody group was also observed compared to that of the PBS group (Fig. 9E), suggesting robust triggering systemic T cell immune responses. To further clarify the underlying mechanisms of the persistently amplifying immune responses, we evaluated the effector memory T cells (CD3^+^CD8^+^CD44^+^CD62L^−^) in the spleen. The percentage of the effector memory T cells in the Fe-PHCN@DOX plus laser plus nanobody group was much higher than that of the PBS group (Fig. 9F, G; Additional file 1: Fig. S32). Consistent with the results of effector memory T cells, the levels of cytokines IFN-γ observed increased after Fe-PHCN@DOX plus laser plus nanobody treatment (Additional file 1: Fig. S33). Our data strongly evidenced that nano-nuclear-reactors together with nanobody could induce a long-term immune memory effect. Encouraged by the strong immune memory against the distant tumors, we subsequently investigated the anti-metastasis effects in a CT26 lung metastasis model. More lung metastasis nodules were observed mice in lung treated with the PBS group, while mice treated with nano-nuclear-reactors together with nanobody group showed less lung metastasis (Fig. 9H). This result also confirmed by H&E staining lung slices (Fig. 9I). Taken together, our results demonstrated that Fe-PHCN@DOX nano-nuclear-reactors together with nanobody could effectively promote persistently systemic antitumor T cell immune responses and inhibit distant and metastatic tumors.

### The safety evaluation

Biosafety is a great concern in anti-tumor therapy before clinical applications. We first evaluated the hemolysis ratio of red cells. The hemolysis ratio of the Fe-PHCN@DOX was less than 4.1% at the maximum concentration (200 μg mL^−1^), suggesting their desirable blood biocompatibility (Additional file 1: Fig. S34). Moreover, the potential biocompatibility of the Fe-PHCN@DOX toward major organs was investigated with histology analysis using H&E staining. No obvious tissue damage or inflammatory lesions were observed in the major organs from all treatment groups (Additional file 1: Fig. S35), indicating the excellent biosafety of the Fe-PHCN@DOX. Furthermore, no obvious abnormal mouse behaviors among all treatment groups were visualized during the treatment process. For liver toxicity, serum levels of AST and ALT in the group of Fe-PHCN@DOX were not obviously increased (Additional file 1: Fig. S36A, B). As one of key inflammatory cytokines, IL-1β was not obviously induced after nano-nuclear reactor treated (Additional file 1: Fig. S36C). Meanwhile, H&E staining data did not show the obvious tissue damage in the major organs (Additional file 1: Fig. S36D). Therefore, the above results revealed that the strategy of using nano-nuclear-reactors mediated therapy was excellent biocompatibility and biosafety, suggesting no obvious side effects in vivo.

## Conclusions

In summary, a targeted nano-nuclear-reactor in combination with nanobody for persistent promoting T cell immune responses for cancer immunotherapy was reported for the first time. The rationally engineered Fe-PHCN@DOX nano-nuclear-reactors that simultaneously reversed the low immunogenicity, hypoxia, and immunosuppressive TME could act as the effective ICD inducers and M1-type macrophage polarizers, and anti-PD-L1 nanobody was served as a PD-L1 immune checkpoint blockader. The Fe-PHCN@DOX nano-nuclear-reactors displayed high nanocatalytic effect, photothermal effect, GSH depletion, and DOX-induced cytotoxicity, thus resulting in effective antitumor efficacy. These reactions resulted in effectively evoking and boosting the ICD effect to promote antigen presentation for DCs maturation, which potentially stimulated T cell activation. Besides, the excellent O_2_ generating capacity of the Fe-PHCN@DOX nano-nuclear-reactors could be applied for tumor hypoxia relief for promoting polarization of M2-type macrophages to M1-type macrophages, thereby effectively enhancing T cell function. Furthermore, anti-PD-L1 nanobody mediated PD-L1 immune checkpoint blockade significantly enhanced the superior T cell immune responses by the blocking of the PD-1/PD-L1 immunosuppressive pathway. Consequently, the combination of three effects effectively realized a persistent T immune response with excellent systemic immune response and immune memory, against primary and distant tumor growth, and tumor metastases. Therefore, this work provided a promising strategy for future clinical applications by evoking persistent T cell immune activation against tumors.

## Materials and methods

### Reagents

Hyaluronic acid (MW 34 kDa) was purchased from Shangdong Freda Biopharm Co., Ltd. Copper (II) chloride (CuCl_2_), Iron (III) chloride (FeCl_3_), doxorubicin hydrochloride (DOX-HCl), sodium hydroxide (NaOH), methylene blue (MB), sodium sulfide nonahydrate (Na_2_S⋅9H_2_O), hydrogen peroxide solution (H_2_O_2_), methoxy PEG thiol (mPEG-SH), and hydrazine were obtained from Sigma-Aldrich (USA). Alexa Fluor 488 Annexin V/PI Cell Apoptosis Kit, 2′,7′-Dichlorofluorescin diacetate (DCF), and Calcein-AM/Propidium Iodide (PI) Staining Kit were purchased from Invitrogen (USA). The Cell Counting Kit (CCK-8) was purchased from Shanghai Yeasen Biotechnology Co., Ltd. The Calcein-AM/Propidium Iodide (PI) Staining Kit was purchased from Invitrogen (USA). Chlorin e6 (Ce6) was purchased from Santa Cruz Biotechnology, Inc. (USA). ATP assay kit, hyaluronidase, and DNase I was purchased from Beyotime Biotechnology. Collagenase (Type IV) powder was purchase from Gibco. RMPI 1640 medium with high concentration of glucose, phosphate buffered saline (PBS) were purchased from Thermo Fisher Scientific. FITC-CD3 monoclonal antibody, PerCP-Cyanine5.5-CD4 monoclonal antibody APC-CD8a monoclonal antibody, FITC-CD11c monoclonal antibody, PE-CD86 monoclonal antibody, APC-CD80 monoclonal antibody, FITC-CD11b monoclonal antibody, PerCP-Cyanine5.5-F4/80 monoclonal antibody, APC-CD206 monoclonal antibody, APC/Cyanine7-CD62L monoclonal antibody, Alexa Fluor@700-CD44 monoclonal antibody, DyLight 488-HIF1α monoclonal antibody were purchased from eBioscience. Anti-calreticulin (CRT) antibody (Alexa Fluor 488) was purchased from Abcam. Anti-HMGB1/PE conjugated antibody was purchased from Bioss. Mouse IL-6 ELISA Kit, mouse TNF α ELISA Kit, mouse and mouse IFN-γ ELISA kit were purchased from eBioscience.

### Synthesis of Fe-PHCN@DOX nano-nuclear-reactors

Fe-PHCN was synthesized according to a previously reported method with minor modifications [26]. In a typical synthesis, CuCl_2_ and FeCl_3_ were dissolved in water containing hyaluronic acid, followed by the addition of 25 mL of NaOH. After stirring for 5 min, hydrazine was added to the above mixture. Na_2_S⋅9H_2_O aqueous stock solution was added to the suspension and heated at 60 °C for 2 h. After centrifugation and washed with deionized water, mPEG-SH was added to the above mixture and mixed with at room temperature for 48 h. Then, Fe-PHCN was obtained by centrifugation and washed with deionized water for three cycles.

For DOX loading, Free DOX (1 mg/mL) was added to the as-prepared Fe-PHCN under strong stirring for 24 h. Subsequently, the Fe-PHCN@DOX nano-nuclear-reactors was collected by centrifugation and washed with water several times to remove any residual free DOX. The free DOX collected from supernatants was quantified by UV–vis spectroscopy measurements. The DOX loading efficiency (LE) can be calculated by the following equation:

$${\text{LE}} \, \left({\%}\right)=\frac{({\text{m}}_{original DOX}-{\mathrm{m}}_{DOX\, in\, supernatants})}{({m}_{original DOX})}\times{100}$$

To prepare Fe-PHCN@DOX/Ce6, Ce6 (5 mg·mL^−1^) was added to the as-prepared Fe-PHCN@DOX under stirring at 37 °C. After 24 h, the Fe-PHCN@DOX/Ce6 was collected by centrifugation and washed with water several times to remove any residual free Ce6.

### In vitro cellular uptake

Flow cytometry was used to quantitatively assess in vitro cellular uptake. CT26 cells were cultured in 24-well plates overnight. Fe-PHCN@DOX nano-nuclear-reactors were then added. After different incubation times, cells were obtained by trypsin digestion and measured by flow cytometry. Meanwhile, cellular Uptake of the Fe-PHCN@DOX nano-nuclear-reactors with/without HA pre-treatment were also measured by flow cytometry.

The internalization and distribution of Fe-PHCN@DOX nano-nuclear-reactors in the cells were observed using a Leica TCS SP5 confocal laser scanning microscope (CLSM). CT26 cells were cultured in 24-well plates overnight. Fe-PHCN@DOX nano-nuclear-reactors were then added with/without HA pre-treatment. After 12 h of incubation, the cells were washed and fixed with paraformaldehyde (4%), stained with DAPI, and observed by CLSM.

### Detection of intracellular ROS

DCF was employed as a fluorescent ROS probe. CT26 cells were cultured in 24-well plates overnight. Fe-PHCN@DOX nano-nuclear-reactors were then added. Subsequently, the cells were stained with DCFH-DA (10 μM) for 30 min. The formed fluorescent matter DCF could be measured by a fluorescence microscope (Olympus IX71, JPN).

### In vitro cytotoxicity of Fe-PHCN@DOX nano-nuclear-reactors

CT26 cells were seeded in 96-well plates at a density of 1 × 10^4^ cells/well overnight. The medium was replaced by the medium containing different materials. Subsequently, the cells were irradiated using the 1064 nm laser (1 W cm^−2^). Then, the cells were further incubated for 4 h at 37 °C under 5% CO_2_. Finally, the cell viabilities were measured by the CCK-8 assay. Meanwhile, Calcein-AM and pyridine iodide (PI) staining reagents were applied to stain the viable/dead cells. The fluorescence was detected using an inverted florescence microscope system (Olympus IX71, JPN). Moreover, Alexa Fluor® 488 Annexin V/PI staining Kit was used for staining apoptotic cells according to the manufacturer’ s instructions. Apoptosis flow cytometric measurements were performed by a flow cytometer (FACS Caliber system, BD Biosciences, Oxford, UK).

### Induced immunologic cell death (ICD) in vitro.

To investigate ICD mechanism in vitro, extracellular release of HMGB1, ATP secretion and surface expression of calreticulin (CRT) were examined. To detect CRT exposure on the surface of cancer cells, CT26 cells were seeded into in 24-well plate at a density of 5 × 10^4^ cells/well and incubated overnight. Then the cells were incubated with different materials for 24 h. The cells were then washed with PBS three times. The cells were collected by trypsin digestion and incubated with Alexa Fluor 488-conjugated anti-CRT antibody for 45 min and then detected by flow cytometry. The data was analysed by the FlowJo software.

ATP secretion was tested using an ATP assay kit. Briefly, CT26 cells were seeded into in 24-well plate at a density of 5 × 10^4^ cells/well and incubated overnight. Then the cells were incubated with different materials for 24 h. Then, the supernatants were collected, and the extracellular ATP content was measured with an ATP Assay Kit according to the manufacture’s instruction (Beyotime).

HMGB1 in the nucleus was determined using immunofluorescence analysis. Briefly, CT26 cells were seeded into the confocal dish plates at a density of 1 × 10^4^ cells/well and incubated overnight. Then the cells were incubated with different materials for 24 h. The cells were then washed with PBS three times. The cells were then fixed with 4% paraformaldehyde for 10 min at room temperature and permeabilized with 0.1% Triton X-100 for 10 min. After blocking with 10% FBS, the cells were incubated with PE-conjugated anti-HMGB1 antibody for 45 min and then stained with DAPI for 10 min, respectively. After staining, images were acquired using a CLSM.

### In vitro DCs maturation

CT26 cells were pretreated with various formulations for 24 h. Afterward, DC2.4 cells were cocultured with pretreated CT26 cells for 24 h. After staining with anti-CD11c- FITC, anti-CD86-PE, and anti-CD80-APC antibodies, DC2.4 cells were detected by flow cytometry. The data was analysed by the FlowJo software.

### In vivo biodistribution of nano-nuclear-reactors

For in vivo fluorescence imaging, tumor xenografts were established by subcutaneous inoculation of 1 × 10^6^ CT26 tumor cells. All mice were used for experiments when the tumors reached the size of around 60 mm^3^. CT26 tumor-bearing mice were intravenously (i.v.) injected with Fe-PHCN@DOX/Ce6 (2.0 mg Ce6/kg), and the obtained fluorescence imaging images on CRI maestro system at 0, 6, 12, and 24 h. The images were unmixed by the Maestro software. Tumors and major organs were collected after 24 h and also subjected to ex vivo imaging.

### In vivo photothermal imaging

For in vivo photothermal imaging, tumor xenografts were established by subcutaneous inoculation of 1 × 10^6^ CT26 tumor cells. All mice were used for experiments when the tumors reached the size of around 60 mm^3^. The tumor-bearing mouse was intravenously injected with the Fe-PHCN@DOX and PBS. After 12 h, the tumor sites were irradiated using a 1064 nm laser (1 W cm^−2^) for 6 min. During the NIR irradiation process, the infrared thermal camera was used to monitor the temperature changes of the tumor sites.

### In vivo antitumor evaluation

The study was conducted according to the guideline of Institutional Anima Care and Use Committee. All animal experimental protocols were approved by the Institutional Animal Care and Use Committee, University of Macau. The approval number was UMARE–041–2020. Tumor xenografts were established by subcutaneous inoculation of 1 × 10^6^ CT26 tumor cells. All mice were used for experiments when the tumors reached the size of around 60 mm^3^. BALB/c mice were randomly divided into five groups (n = 5, in each group) including: (a) PBS; (b) Fe-PHCN; (c) Fe-PHCN@DOX; (d) Fe-PHCN@DOX plus laser irradiation; and (e) Fe-PHCN@DOX plus laser irradiation plus anti-PD-L1 nanobody. After 8 days’ tumor inoculation, tumor-bearing mice were intravenously injected with nano-nuclear-reactors on day 1. At 12 h post-injection, the tumors (d and e groups) were irradiated with a 1064 nm laser (1 W cm^−2^) for 6 min. 75 μg anti-PD-L1 nanobody was locally injected in tumor sites (e group) on day 2, 4, and 6. Tumor growth and weight were monitored every 3 days by measurement with a digital display caliper, where tumor volumes were calculated as follows: (width^2^ × length)/2. Then, the collected tumors were fixed immediately in 10% paraformaldehyde solution, followed by standard dehydration and paraffin embedding. The embedded tissues werethen sectioned into 10 μm slices and then subjected to standard H&E and TUNEL staining for histological analysis. Treatment-induced CRT exposure and HIF-1α expression at the tumor site were examined by immunofluorescence.

### In vivo DCs maturation

To detect DC maturation in vivo, the tumor-draining lymph nodes (LNs) were collected at 7th day’s post-treatment. A single-cell suspension was obtained by grinding lymph nodes and passed though the 70 μm cell strainer. Then, the frequency of matured DC in the LNs was then investigated by flow cytometry after staining with anti-CD11c-FITC, anti-CD86-PE, and anti-CD80-APC antibodies (gating on CD11c^+^ cells). The data was analysed by the FlowJo software.

### In vivo T cell infiltration and macrophages polarization

To detect T cell infiltration in vivo, the tumors were harvested collected at 7th day’s post-treatment, cut into small pieces, and immersed in a solution of 1.0 mg/mL collagenase IV, hyaluronidase (100 U) and 0.2 mg/mL DNase I for 60 min at 37 °C. After that, a single-cell suspension was obtained by homogening tumor cells and passed though the 70 μm cell strainer. The cell suspension was then incubated with antibodies. The cell suspension was stained with anti-CD3-FITC, anti-CD4-PerCP-Cyanine5.5, and anti-CD8α-APC, and then detected by flow cytometry. Meanwhile, to detect the polarization of macrophages, the cell suspension was stained with anti-F4/80-PerCP-Cyanine5.5, anti-CD86-PE, anti-CD11b-FITC, and anti-CD206-APC, and then detected by flow cytometry. The data was analysed by the FlowJo software.

### Cytokine detection

Serum samples were isolated and collected from mice after various treatments at 7th day’s post-treatment. After centrifugation, Tumor necrosis factors (TNF-α), IFN-γ, and IL-6 in serum were analyzed with ELISA kits.

### The abscopal effect study

To investigate the abscopal effect of nano-nuclear-reactors, 1 × 10^6^ CT26 cells were seeded on the left/right side of the same mice on day -8 and 0. After the tumor volume reach about 60 mm^3^, and tumor-bearing mice were randomly divided into two groups: (a) PBS; (b) Fe-PHCN@DOX plus laser plus anti-PD-L1 nanobody. Then, tumor-bearing mice were intravenously injected with nano-nuclear-reactors on day 1. Then, the primary tumors were performed under 1064 nm laser irradiation 12 h post-injection, and anti-PD-L1 nanobody was intratumorally injected on day 2, 4, and 6. The distant tumor growth and weight were monitored every 3 days by measurement with a digital display caliper, where tumor volumes were calculated as follows: (width^2^ × length)/2.

To evaluate the infiltrating CD8 + T cell in distant tumors, these tumors were surgically excised at 7th day’s post-treatment for immunofluorescence staining. Next, frozen sections of the tumors were prepared with optimum cutting temperature (OCT) compound. Then, the staining was done by incubation with mouse APC-anti-CD3 FITC-CD8 monoclonal antibody. Cell nuclei were stained with DAPI. The stained tumor sections were imaged under the confocal fluorescence microscopy (Leica SP5).

The immune memory effect of CD8^+^ T cells was examined after the final treatment. The spleen were collected from BALB/c mice at 27th day’s post-treatment. A single-cell suspension was obtained by grinding lymph nodes and passed though the 70 μm cell strainer. After staining with anti-CD3-FITC, anti-CD8α-APC, anti-CD44-Alexa Fluor@700, and anti-CD62L-APC/Cyanine7 antibodies, the frequency of the effector memory T cells was measured by flow cytometry. The data was analysed by the FlowJo software.

### In vivo lung metastases evaluation

To evaluate the efficacy of nano-nuclear-reactors in inhibiting lung metastases, a CT26-bearing mice model was established. Tumor xenografts were established by subcutaneous inoculation of 1 × 10^6^ CT26 tumor cells in the right flank of mice on day -8. After the tumor volume reach about 60 mm^3^, and tumor-bearing mice were randomly divided into two groups: a) PBS; b) Fe-PHCN@DOX plus laser plus anti-PD-L1 nanobody. Then, tumor-bearing mice were intravenously injected with nano-nuclear-reactors on day 1. Then, the primary tumors were performed under 1064 nm laser irradiation 12 h post-injection, and anti-PD-L1 nanobody was intratumorally injected on day 2, 4, and 6. Then, the Balb/c mice were injected CT26 cells intravenously after various treatments. After 18 days, the mice were sacrificed and lung tissue from each group of mice was stained with picric acid to detect the metastatic nodules. The collected lung were fixed and then subjected to standard H&E staining for histological analysis.

### In vivo biocompatible evaluation

The major organs (heart, liver, spleen, lung, and kidney) were harvested from the treated Balb/c mice at 24th day’s post-treatment and fixed using paraformaldehyde. Tissue samples were then embedded in paraffin, sliced, and stained using H&E. The histological sections were observed under an optical microscope.

### Statistical analysis

All statistical analyses were performed using GraphPad Prism software. Significant differences were calculated using the Log-rank (Mantel-Cox) test. P values are represented as: * p < 0.05, ** p < 0.01, and *** p < 0.001.

## Supplementary Information

**Additional file 1: Methods. Fig. S1.** SEM image of Fe-PHCN. Scale bar: 1 μm. **Fig. S2.** TEM image of Fe-PHCN@DOX. The scale bar represents 100 nm. **Fig. S3.** Size of Fe-PHCN by dynamic light scattering (DLS) measurement. **Fig. S4.** Energy-dispersive X-ray spectroscopy (EDX) of Fe-PHCN. **Fig. S5.** Zeta potential of Fe-HCN and Fe-PHCN by DLS measurement. **Fig. S6.** FT-IR spectra of Fe-HCN and Fe-PHCN. **Fig. S7.** UV-vis absorbance spectra of the Fe-PHCN in NIR I and NIR II region. **Fig. S8.** Digital photograph of DOX, Fe-PHCN, and Fe-PHCN@DOX. **Fig. S9.** The size changes of Fe-PHCN@DOX in water, PBS, and DMEM medium containing 10% FBS for 6 days. **Fig. S10.** UV−vis spectra (A) and photographs (B) of MB aqueous solution under different concentrations of Fe-PHCN. **Fig. S11.** Flow cytometry analysis of uptake level of the Fe-PHCN@DOX in the CT26 cells with/without HA treatment. **Fig. S12.** Fluorescence images of DCF-stained CT26 cells under PHCN and Fe-PHCN treatment. **Fig. S13.** Flow cytometry gating strategy for the analysis of Annexin V-FITC/PI co-staining cell apoptosis. **Fig. S14.** Coomassie blue staining was performed after SDS-PAGE running: M represents marker; 1 represents anti-PD-L1 antibody; 2 represents anti-PD-L1 nanobody. **Fig. S15.** Confocal microscopy images (A) and fluorescence intensity (B) of anti-PD-L1 nanobodies and antibody distribution in tumor sections after injection. The scale bar is 50 μm. **Fig. S16.** The binding activity of PD-L1 receptor on CT26 cells with BSA, anti-PD-L1 nanobody (Nab), and anti-PD-L1 antibody (ab) was measured by flow cytometry. **Fig. S17.** The levels of cytokines released from T cells after different treatment. **Fig. S18.** The quantification (A) and percentage (B) of CD69+CD8+ T cells after different treatment. **Fig. S19.** The quantification (A) and percentage (B) of M1-type macrophages (CD11b+F4/80+CD86+CD206-) and M2-type macrophages (CD11b+F4/80+CD86-CD206+) in vitro after different treatment by flow cytometry. **Fig. S20.** Flow cytometry gating strategy for the analysis of DCs maturation in vitro. **Fig. S21.** ELISA analysis of the levels of cytokines TNF-⍺ secreted by DC2.4 cells in the medium. **Fig. S22.** The GSH depletion in tumor tissues after under different concentrations of Fe-PHCN@DOX. **Fig. S23.** Tumor growth curve of CT26 tumor-bearing mice after different treatments in 12 days. **Fig. S24.** Flow cytometry gating strategy for the analysis of DCs maturation in vivo. **Fig. S25.** Flow cytometry gating strategy for the analysis of M1-TAMs and M2-TAMs in vivo. **Fig. S26.** Flow cytometry gating strategy for the analysis of CD8 cells and CD4 cells in vivo. **Fig. S27.** The quantification of CD4+ helper T (CD3+CD4+) cells by flow cytometric analyses after various treatments. **Fig. S28.** The quantification of IFN-γ+ CD8+ T cells (A), Granzyme B+ CD8+ T cells (B) and Perforin+ CD8+ T cells (C) by flow cytometric analyses after different treatments. **Fig. S29.** The quantification of IFN-γ+ CD4+ T cells (A) and TNF-α+ CD4+ T cells (B) by flow cytometric analyses after different treatments. **Fig. S30.** The quantification of Treg cells (CD3+CD4+Foxp3+) by flow cytometric analyses after different treatments. **Fig. S31.** The quantification (A) and percentage (B) of infiltrating T cells in tumors by flow cytometric analyses after different treatment. **Fig. S32.** Flow cytometry gating strategy for the analysis of effector memory T cells in vivo. **Fig. S33.** ELISA analysis of the levels of cytokines IFN-γ in serum of mice after PBS and Fe-PHCN@DOX plus laser plus nanobody treatments. **Fig. S34.** Hemolytic percent of red blood cells incubated with Fe-PHCN@DOX at various concentrations. **Fig. S35.** H&E-stained images of heart, liver, spleen, lung, and kidney of the mice treated with PBS and Fe-PHCN@DOX plus laser plus nanobody. Scale bar stands for 100 μm. **Fig. S36.** Serum levels of ALT (A), AST (B), and IL-1β (C) were measured after the Fe-PHCN@DOX treatments. (D) H&E staining of the major organs including heart, liver, spleen, lung, and kidney after treatments. Scale bar stands for 200 μm.

## Data Availability

The datasets used and/or analyzed during the current study are available from the corresponding author on reasonable request.

## Abbreviations

TME: Tumor microenvironment
DOX: Doxorubicin
PHCN: HA@Cu_2−X_S-PEG
ICD: Immunogenic cell death
TAMs: Tumor-associated macrophages
DCs: Dendritic cells
PD-1: Programmed death-1
PD-L1: Programmed death-ligand 1
TAA: Tumor-associated antigen
DAMPs: Damage-associated molecular patterns
NCT: Nanocatalytic therapy
ROS: Reactive oxygen species
NIR: Near-infrared
HA: Hyaluronic acid
H_2_O_2_: Hydrogen peroxide
•OH: Hydroxyl radicals
Nab: Nanobody
MB: Methylene blue
DCF: 2ʹ,7ʹ-Dichlorofluorescein diacetate
CRT: Calcium netting protein
ATP: Adenosine triphosphate
HMGB1: High mobility group box 1
Ce6: Chlorin e6
HIF-1α: Hypoxia inducible factors-1 alpha
