# Supplementary material for: Synergistic combination of targeted nano-nuclear-reactors and anti-PD-L1 nanobodies evokes persistent T cell immune activation for cancer immunotherapy
Source: J Nanobiotechnology. 2022 Dec 10;20:521. doi: 10.1186/s12951-022-01736-8 (PMC9741809; doi:10.1186/s12951-022-01736-8)
Supplement: Supplementary file 1 — Additional file 1: Methods. Fig. S1. SEM image of Fe-PHCN. Scale bar: 1 μm. Fig. S2. TEM image of Fe-PHCN@DOX. The scale bar represents 100 nm. Fig. S3. Size of Fe-PHCN by dynamic light scattering (DLS) measurement. Fig. S4. Energy-dispersive X-ray spectroscopy (EDX) of Fe-PHCN. Fig. S5. Zeta potential of Fe-HCN and Fe-PHCN by DLS measurement. Fig. S6. FT-IR spectra of Fe-HCN and Fe-PHCN. Fig. S7. UV-vis absorbance spectra of the Fe-PHCN in NIR I and NIR II region. Fig. S8. Digital photograph of DOX, Fe-PHCN, and Fe-PHCN@DOX. Fig. S9. The size changes of Fe-PHCN@DOX in water, PBS, and DMEM medium containing 10% FBS for 6 days. Fig. S10. UV−vis spectra (A) and photographs (B) of MB aqueous solution under different concentrations of Fe-PHCN. Fig. S11. Flow cytometry analysis of uptake level of the Fe-PHCN@DOX in the CT26 cells with/without HA treatment. Fig. S12. Fluorescence images of DCF-stained CT26 cells under PHCN and Fe-PHCN treatment. Fig. S13. Flow cytometry gating strategy for the analysis of Annexin V-FITC/PI co-staining cell apoptosis. Fig. S14. Coomassie blue staining was performed after SDS-PAGE running: M represents marker; 1 represents anti-PD-L1 antibody; 2 represents anti-PD-L1 nanobody. Fig. S15. Confocal microscopy images (A) and fluorescence intensity (B) of anti-PD-L1 nanobodies and antibody distribution in tumor sections after injection. The scale bar is 50 μm. Fig. S16. The binding activity of PD-L1 receptor on CT26 cells with BSA, anti-PD-L1 nanobody (Nab), and anti-PD-L1 antibody (ab) was measured by flow cytometry. Fig. S17. The levels of cytokines released from T cells after different treatment. Fig. S18. The quantification (A) and percentage (B) of CD69+CD8+ T cells after different treatment. Fig. S19. The quantification (A) and percentage (B) of M1-type macrophages (CD11b+F4/80+CD86+CD206-) and M2-type macrophages (CD11b+F4/80+CD86-CD206+) in vitro after different treatment by flow cytometry. Fig. S20. Flow cytometry gating strategy for th [file 12951_2022_1736_MOESM1_ESM.docx]

**Synergistic combination of targeted nano-nuclear-reactors and anti-PD-L1 nanobodies evokes persistent T cell immune activation for cancer immunotherapy**

Lipeng Zhu ^1†^*, Junnan Li ^2†^, Ziang Guo ^2^, Hang Fai Kwok ^2^ and Qi Zhao ^2^*

^†^Lipeng Zhu and Junnan Li contributed equally to this work.

*Correspondence: [zhuleaper@csu.edu.cn](mailto:zhuleaper@csu.edu.cn); [qizhao@um.edu.mo](mailto:qizhao@um.edu.mo)

^1^ School of Life Sciences, Xiangya School of Medicine, Central South University, Changsha 510006, China.

^2^ Cancer Centre, Institute of Translational Medicine, Faculty of Health Sciences, University of Macau, Macau SAR 999078, China.

**Methods**

**Cell lines and animals**

CT26 cancer cells were purchased from the American Type Culture Collection and were cultured in RMPI 1640 medium containing 10% FBS and 1% antibiotics at 37 °C in a CO_2_ atmosphere. DC2.4 cells were obtained from the cell bank of the Faculty of Health Science, University of Macau, and cultured in RMPI 1640 medium containing 15% FBS and 1% antibiotics at 37 °C in a CO_2_ atmosphere. Balb/c mice (4-6 weeks, ～20 g) were purchased from Animal Research Core of University of Macau. The study was conducted according to the guideline of Institutional Animal Care and Use Committee. All animal procedures were performed following an approved protocol (UMARE-041-2020) by the University of Macau Animal Ethics Committee.

**Characterization of Fe-PHCN@DOX**

The particle size and zeta potential of the nanoparticles could be measured using a Nano-ZS ZEN3600 (Malvern). (UV-vis)-NIR spectral data were determined by using a PerkinElmer Lambda UV spectrophotometer. The morphologies of the products were determined using a field emission scanning electron microscope (FE-SEM, S-4800, Hitachi). Low-/high-resolution transmission electron microscopy (TEM) was carried out and the composition of samples was determined on an FEI Tecnai G2S-Twin instrument equipped with an energy-dispersive X-ray (EDX) spectrometer. The oxygen concentration in water was monitored using an Ysi Dissolved Oxygen Meter. Confocal microscopy images were acquired on a Nikon A1R fluorescence microscope. Flow cytometry analysis was conducted with BD Beckman Coulter flow cytometer and FlowJo Software 10.0 (TreeStar, Ashland, OR).

**Stability.**

To assess the stability of the Fe-PHCN@DOX nano-nuclear-reactors in vitro, their size was measured after different time periods in water, phosphate-buffered saline (PBS), and DMEM containing 10% FBS.

**Photothermal performance**.

For photothemal effect, the temperature changes of the Fe-PHCN nano-nuclear-reactors and PBS were measured under a 1064 nm laser (1 W cm^-2^, 6 min) using an infrared thermal imaging camera (Fluke). The temperature changes of the Fe-PHCN nano-nuclear-reactors at a series of different concentrations under NIR laser irradiation were monitored.

**pH and NIR responsive drug release**

To determine pH-responsive drug release, the Fe-PHCN@DOX nano-nuclear-reactors were treated in PBS buffer at different pH values (7.4 and 5.2) and shaken gently. To evaluate NIR sensitivity of the nano-nuclear-reactors, Fe-PHCN@DOX nano-nuclear-reactors were repeatedly exposed to a NIR laser irradiation (1 W cm^-2^). Subsequently, the released free DOX was collected at time point and quantified by UV–vis spectra.

**Nanocatalytic properties**

For the extracellular •OH generation detection, MB solution was mixed with Fe-PHCN and H_2_O_2_ at 37 ^o^C for 30 min. Then the absorbance change of •OH-induced MB degradation was monitored at 665 nm. The MB solutions treated with Fe-PHCN or H_2_O_2_ alone were used as control groups. In addition, the •OH generation was measured under H_2_O_2_ at a series of different concentrations and further tested with/without the 1064 nm laser irradiation.

**Catalase-like activity**

For the catalase-like activity to produce O_2_ capacity, the H_2_O_2_ was added into Fe-PHCN aqueous solution. Then, the generated concentration of O_2_ was monitored by a portable dissolved oxygen meter. The Fe-PHCN solutions without H_2_O_2_ treatment were used as control groups.

**GSH depletion activity**

The depletion of GSH was measured by UV−visible spectroscopy. A DTNB PBS solution (3.0 mg·mL^-1^) and GSH aqueous solution (10 mM) were added into Fe-PHCN aqueous solution at different concentrations, respectively. After that, the mixtures were maintained at 25 °C under magnetic stirring for 1 h and the absorbance of the solution was measured by UV−vis spectroscopy.

**Expression and purification of anti-mouse PD-L1 nanobody**

The OmpA signal peptide was add to the N-terminal, Flag tag and a hexahistidine (His6) tag was added to the C-terminal of anti- mouse PD-L1 VHH. Then it was subcloned into pComb3x vector. It's expression was performed in Escherichia coli HB2151 bacterial culture at 30 ℃ for 16 h with 0.5mM IPTG. The bacterial were harvested and lysed by Polymyxin B (Aladdin) at 30℃ for 0.5h. Supernatant was obtained by centrifugation at 8000 rpm for 10 min and loaded over Ni-NTA as manual described (GIQGEN). Resin was washed by washing buffer [10 mM PBS (pH 7.2~7.4), 300 mM NaCl and 18 mM imidazole] and protein were eluted in elution buffer [10 mM PBS (pH 7.4), 300 mM NaCl and 200 mM imidazole]. The collected pure fractions were immediately buffer-exchanged into phosphate buffered saline solution (PBS) (Sigma) and concentrated using an Amicon ultra centrifugal concentrator (Millipore) with a molecular weight cut-off of 3 kDa. Purity was estimated to be over 95% by SDS-polyacrylamide gel electrophoresis, and protein concentration was measured using the NanoDrop OneC spectrophotometer.

**Measurement of anti-mouse PD-L1 nanobody’s molecular weight**

To measure molecular weights of the anti-mouse PD-L1 nanobody, 2 μg anti-mouse PD-L1 nanobody and anti-mouse PD-L1 IgG (Biolegend) were run on SDS-PAGE gel and stained with Coomassie blue. The molecular weights of each sample were measured with pre-stained protein marker.

**The binding activity of anti-PD-L1 nanobody/PD-L1**

Enzyme-linked immunosorbent assay (ELISA) was used to evaluate the anti-PD-L1 nanobody/PD-L1 binding activity. ELISA plate was coated with mouse PD-L1 antigen (50 ng per well, Biolegend), blocked by 0.2% PBST containing 2.5% skim milk for 0.5h at 37℃. Antigen were incubated for 0.5 h at 37℃ by different dilutions anti-mouse PD-L1 nanobody after washed by 0.2% PBST. Bound antibodies were detected with the secondary mouse anti-flag-HRP antibody (1:5,000 dilution). The 2,20-azino-bis-(3-ethylbenzthiazoline-6-sulfonic acid) substrate (Beyotime) was added, and the reaction was read at 450 nm after ended by 2M H_2_SO_4_.

**Intracellular GSH measurement**

The Intracellular GSH was measured by the GSH and GSSG Assay Kit (Beyotime). CT26 cells were cultured in 24-well plates and treated with PBS and Fe-PHCN@DOX with different concentrations. After incubating for 6 h, the cells were washed with PBS for 3 times. Then, the cells were collected with centrifugation, and the supernatant was discarded. The cell precipitates were resuspended in protein remover M, subjected to 3 cycles of freezing−thawing, and then centrifugated at 10000 g for 10 min at 4 °C. The supernatant was reserved for GSH and GSSH assay according to the manufacturer’s protocol.

**Cell cycle arrest**

CT26 cells were seeded in 6-well plates at a density of 2×10^5^ cells/well overnight. After exposed to different treatments for 24 h, Cells were collected and washed with PBS. Then those cells were fixed with cold 70% ethanol overnight at −20 °C. After fixing, cells were washed with PBS and re-suspended in 200 μg mL^−1^ DNase-free RNase A (Thermo Fisher, 12091021), incubating for 30 min in 37°C. After washing with PBS, the cells were suspended in 0.5 mL PBS, containing propidium iodide and 0.1% Triton X-100 for 15 min at room temperature. DNA content was analyzed for cell cycle by a flow cytometer (FACS Caliber system, BD Biosciences, Oxford, UK). FlowJo software (Tree Star, Inc.) was used to qualify cell cycle distribution.

**Measurement of intracellular generation of O_2_.**

[Ru(dpp)_3_]Cl_2_ (RDPP) was used to detect the intracellular production of O_2_. CT26 cells were seeded into culture dishes for 24 h (37 °C in 5% CO_2_). Then, RDPP medium solution (1 μM) was added to the cells for another 4 h. After that, the CT26 cells were washed with PBS and further incubated with Fe-PHCN. Finally, the cells were washed by PBS, and the strength of green fluorescence was obtained using an inverted florescence microscope system (Olympus IX71, JPN).

**The hemolysis ratio evaluation**

To evaluate the blood biocompatibility of the Fe-PHCN@DOX nano-nuclear-reactors, the hemolysis ratio of red cells was used to be evaluated [[1](#_ENREF_1)]. The fresh blood was centrifuged and then diluted with PBS. The red blood cells were mixed with Fe-PHCN@DOX nano-nuclear-reactors at different concentrations and incubated at 37 °C for 4 h, and then centrifuged. The release of hemoglobin was determined at 540 nm by a microplate reader. Red blood cells with PBS treatment and Triton-X were considered as the negative control and positive control, respectively. Percentage of hemolysis was calculated using the following equations:

$$\text{Hemolytic}\text{ }\left( \text{\%} \right)\text{ }$$

$$\text{= }\frac{(absorbance for polyplexes treatment - absorbance for PBS treatment)}{(absorbance for Triton-X treatment - absorbance for PBS treatment)}\text{ × 100}$$

**Supplementary figures**

Fig. S1. SEM image of Fe-PHCN. Scale bar: 1 μm.

Fig. S2. TEM image of Fe-PHCN@DOX. The scale bar represents 100 nm.

Fig. S3. Size of Fe-PHCN by dynamic light scattering (DLS) measurement.

Fig. S4. Energy-dispersive X-ray spectroscopy (EDX) of Fe-PHCN.


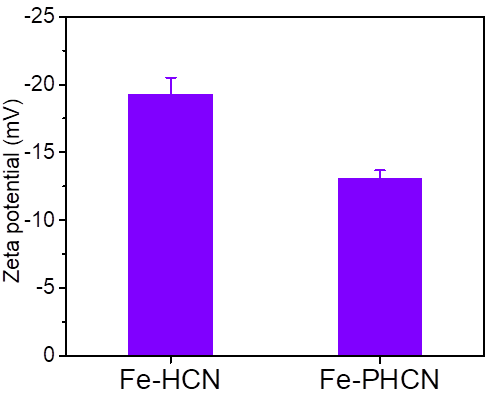


Fig. S5. Zeta potential of Fe-HCN and Fe-PHCN by DLS measurement.

Fig. S6. FT-IR spectra of Fe-HCN and Fe-PHCN.

Fig. S7. UV-vis absorbance spectra of the Fe-PHCN in NIR I and NIR II region.

Fig. S8. Digital photograph of DOX, Fe-PHCN, and Fe-PHCN@DOX.

Fig. S9. The size changes of Fe-PHCN@DOX in water, PBS, and DMEM medium containing 10% FBS for 6 days.

Fig. S10. UV−vis spectra (A) and photographs (B) of MB aqueous solution under different concentrations of Fe-PHCN.

Fig. S11. Flow cytometry analysis of uptake level of the Fe-PHCN@DOX in the CT26 cells with/without HA treatment.

Fig. S12. Fluorescence images of DCF-stained CT26 cells under PHCN and Fe-PHCN treatment.

Fig. S13. Flow cytometry gating strategy for the analysis of Annexin V-FITC/PI co-staining cell apoptosis.

Fig. S14. Coomassie blue staining was performed after SDS-PAGE running: M represents marker; 1 represents anti-PD-L1 antibody; 2 represents anti-PD-L1 nanobody.

Fig. S15. Confocal microscopy images (A) and fluorescence intensity (B) of anti-PD-L1 nanobodies and antibody distribution in tumor sections after injection. The scale bar is 50 μm.

Fig. S16. The binding activity of PD-L1 receptor on CT26 cells with BSA, anti-PD-L1 nanobody (Nab), and anti-PD-L1 antibody (ab) was measured by flow cytometry.

Fig. S17. The levels of cytokines released from T cells after different treatment.

Fig. S18. The quantification (A) and percentage (B) of CD69^+^CD8^+^ T cells after different treatment.

Fig. S19. The quantification (A) and percentage (B) of M1-type macrophages (CD11b^+^F4/80^+^CD86^+^CD206^-^) and M2-type macrophages (CD11b^+^F4/80^+^CD86^-^CD206^+^) *in vitro* after different treatment by flow cytometry.

Fig. S20. Flow cytometry gating strategy for the analysis of DCs maturation *in vitro*.

Fig. S21. ELISA analysis of the levels of cytokines TNF-⍺ secreted by DC2.4 cells in the medium.

Fig. S22. The GSH depletion in tumor tissues after under different concentrations of Fe-PHCN@DOX.

Fig. S23. Tumor growth curve of CT26 tumor-bearing mice after different treatments in 12 days.

Fig. S24. Flow cytometry gating strategy for the analysis of DCs maturation *in vivo*.

Fig. S25. Flow cytometry gating strategy for the analysis of M1-TAMs and M2-TAMs *in vivo*.

Fig. S26. Flow cytometry gating strategy for the analysis of CD8 cells and CD4 cells *in vivo*.

Fig. S27. The quantification of CD4^+^ helper T (CD3^+^CD4^+^) cells by flow cytometric analyses after various treatments.

Fig. S28. The quantification of IFN-γ^+^ CD8^+^ T cells (A), Granzyme B^+^ CD8^+^ T cells (B) and Perforin^+^ CD8^+^ T cells (C) by flow cytometric analyses after different treatments.

Fig. S29. The quantification of IFN-γ^+^ CD4^+^ T cells (A) and TNF-α^+^ CD4^+^ T cells (B) by flow cytometric analyses after different treatments.

Fig. S30. The quantification of Treg cells (CD3^+^CD4^+^Foxp3^+^) by flow cytometric analyses after different treatments.

Fig. S31. The quantification (A) and percentage (B) of infiltrating T cells in tumors by flow cytometric analyses after different treatment.

Fig. S32. Flow cytometry gating strategy for the analysis of effector memory T cells *in vivo*.

Fig. S33. ELISA analysis of the levels of cytokines IFN-γ in serum of mice after PBS and Fe-PHCN@DOX plus laser plus nanobody treatments.

Fig. S34. Hemolytic percent of red blood cells incubated with Fe-PHCN@DOX at various concentrations.

Fig. S35. H&E-stained images of heart, liver, spleen, lung, and kidney of the mice treated with PBS and Fe-PHCN@DOX plus laser plus nanobody. Scale bar stands for 100 μm.

Fig. S36. Serum levels of ALT (A), AST (B), and IL-1β (C) were measured after the Fe-PHCN@DOX treatments. (D) H&E staining of the major organs including heart, liver, spleen, lung, and kidney after treatments. Scale bar stands for 200 μm.

1. Zhu L, Li P, Gao D, Liu J, Liu Y, Sun C, et al. pH-sensitive loaded retinal/indocyanine green micelles as an “all-in-one” theranostic agent for multi-modal imaging in vivo guided cellular senescence-photothermal synergistic therapy. Chem Comm. 2019; 55(44): 6209-12.
